# Candidate Glycoprotein Biomarkers for Canine Visceral Hemangiosarcoma and Validation Using Semi-Quantitative Lectin/Immunohistochemical Assays

**DOI:** 10.3390/vetsci8030038

**Published:** 2021-02-27

**Authors:** Patharee Oungsakul, Eunju Choi, Alok K. Shah, Ahmed Mohamed, Caroline O’Leary, David Duffy, Michelle M. Hill, Helle Bielefeldt-Ohmann

**Affiliations:** 1 School of Veterinary Science, The University of Queensland, Gatton, QLD 4343, Australia; patharee.oungsakul@uq.net.au (P.O.); c.oleary1000@gmail.com (C.O.); 2 The University of Queensland Diamantina Institute, The University of Queensland, Woolloongabba, QLD 4102, Australia; eachoi@ucdavis.edu (E.C.); akshahpharma@gmail.com (A.K.S.); David.Duffy@qimrberghofer.edu.au (D.D.); Michelle.Hill@qimrberghofer.edu.au (M.M.H.); 3 Department of Pathology, Microbiology & Immunology, School of Veterinary Medicine, University of California, Davis, CA 95616, USA; 4 QIMR Berghofer Medical Research Institute, Herston, QLD 4006, Australia; mohamed.a@wehi.edu.au; 5 School of Chemistry & Molecular Biosciences, The University of Queensland, St. Lucia, QLD 4072, Australia

**Keywords:** hemangiosarcoma, dog, glycoprotein, lectin, histochemistry, H-score, complement C7

## Abstract

Visceral hemangiosarcoma (HSA) is one of the more frequent cancers in dogs and has a high metastatic rate and poor prognosis, as clinical signs only become apparent in advanced stages of tumor development. In order to improve early and differential diagnostic capabilities and hence, prognosis for dogs with HSA, two types of biomarker are needed: a point-of-care diagnostic biomarker and a prognostic biomarker—preferentially based on samples obtained with minimally invasive methods. In this study, we applied a lectin magnetic bead array-coupled tandem mass spectrometry (LeMBA-MS/MS) workflow through discovery and validation phases to discover serum glycoprotein biomarker candidates for canine HSA. By this approach, we found that *Datura stramonium* (DSA), wheat germ agglutinin (WGA), *Sambucus nigra* (SNA), and *Pisum sativum* (PSA) lectins captured the highest number of validated candidate glycoproteins. Secondly, we independently validated serum LeMBA-MS/MS results by demonstrating the in situ relationship of lectin-binding with tumor cells. Using lectin-histochemistry and immunohistochemistry (IHC) for key proteins on tissues with HSA and semi-quantitation of the signals, we demonstrate that a combination of DSA histochemistry and IHC for complement C7 greatly increases the prospect of a more specific diagnosis of canine HSA.

## 1. Introduction

The visceral form of hemangiosarcoma (HSA) remains one of the more frequent, and invariable fatal, cancers in dogs, where it most commonly develops in spleen, liver, and heart. There are no specific clinical signs until the tumor ruptures, resulting in massive blood loss, or metastasizes to organs such as brain, lung, or locally within the body cavities. Sudden death or euthanasia are often the consequence. In clinical practice, the mass, along with part or the entire affected organ, especially the spleen, may be surgically removed. However, the survival time of dogs diagnosed with HSA is less than a year [1,2]. A confirmatory diagnosis of the cancer relies on histopathological assessment of tissue samples and yet, no definite prognosis can be derived despite several evaluation methods or tissue markers applied [3].

To improve the quality of life for dogs with HSA, two types of biomarker are needed: a point-of-care diagnostic biomarker for early and differential diagnosis, and a prognostic biomarker to assess survival prospects after surgical or other interventions and preferentially using non- or minimally invasive approaches for sample procurement. The goal of HSA biomarker studies is to detect HSA cell products, such as proteins, in the blood and this is a major focus of many current projects [4,5,6]. Among potential serum protein biomarkers for canine visceral HSA reported in the literature, serum collagen XXVII alpha-1, big endothelin-1 (ET-1), and procoagulant tissue factor (TF) are considered promising candidates [6,7,8]. While serum ferritin, thymidine kinase 1 (TK-1), alpha-1 acid glycoprotein (AGP), plasma cardiac troponin I (cTnI), and vascular endothelial growth factor (VEGF) are also important biomarkers for several vascular diseases, they are less specific for HSA [2,4,9,10,11].

Aberrant glycosylation has been associated with multiple aspects of cancer, and many existing cancer markers are glycosylated proteins [12]. Lectins are naturally occurring proteins that are widely used to recognize different glycosylated forms of proteins. Previously, our group developed a lectin magnetic bead array-coupled tandem mass spectrometry (LeMBA-MS/MS) workflow and demonstrated its application in human, mouse, and canine serum samples [13]. Here, we applied a LeMBA-MS/MS workflow through discovery and validation phases to discover serum glycoprotein biomarker candidates in canine HSA. By this approach we found that *Datura stramonium* (DSA), wheat germ agglutinin (WGA), *Sambucus nigra* (SNA), and *Pisum sativum* (PSA) lectins captured the highest number of validated candidate glycoproteins. Finally, we independently validated the serum LeMBA-MS/MS results by demonstrating the in situ relationship of lectin-binding with tumor cells. This was achieved by (i) lectin histochemistry with identification of the location of the glycoproteins in spleen tissues, either in normal or cancer tissue; and (ii) quantification of the lectin-bound glycoproteins and key proteins (maltase-glucoamylase, vitronectin, complement C7) in the tissues using a semi-quantitative lectin/immunohistochemical signal scoring method. In addition, we assessed the potentials of those ligands to be applied as diagnostic histochemical marker(s) for canine HSA compared to a routinely used immunohistochemical endothelial cell marker, platelet endothelial cell adhesion molecule 1 (PECAM-1, CD31). Here, we report that a combination of DSA lectin histochemistry and complement C7 immunohistochemistry may afford a more specific diagnostic tool for canine HSA.

## 2. Materials and Methods

### 2.1. Samples for Discovery Proteomics Acquisition and Analysis

A total of 20 serum samples were used for the discovery phase, 10 HSA and 10 normal (Table S1A). Of the 10 HSA samples, eight were purchased from the Colorado State University (CSU) Sample Bank (Fort Collins, CO, USA) and two samples were collected from the Brisbane Veterinary Specialist Centre (BVSC), Albany Creek, Australia. Serum samples from normal, cancer-free dogs were collected from the University of Queensland Small Animal Teaching Hospital.

All samples were collected with ethical approval obtained from the University of Queensland Animal Ethics Committee (permit nos. SVS/470/12/AACF and SVS/438/15/Kibble/CRF). For the proteomics validation (Table S1), 43 serum samples from dogs with HSA and 20 serum samples from dogs with an HSA-like splenic mass, which included all other splenic masses excluding HSA, were purchased from the CSU Sample Bank. Thirty serum samples from normal dogs were collected at the University of Queensland Small Animal Teaching Hospital. Twelve HSA and 13 HSA-like samples were collected from the University of Queensland Small Animal Teaching Hospital, Brisbane Veterinary Specialist Centre, and Queensland Veterinary Specialists (QVS).

### 2.2. Discovery Proteomics

LeMBA-MS/MS was conducted using 20 individual lectins exactly as previously described [13]. Briefly, individual lectins (AAL, *Aleuria aurantia* lectin; BPL, *Bauhinia purpurea* lectin; ConA, Concanavalin A from *Canavalia ensiformis*; DSA, *Datura stramonium* agglutinin; ECA, *Erythrina cristagalli* agglutinin; EPHA, Erythroagglutinin *Phaseolus vulgaris*; GNL, *Galanthus nivalis* lectin; HAA, *Helix aspersa* agglutinin; HPA, *Helix pomatia* agglutinin; JAC, Jacalin from *Artocarpus integrifolia*; LPHA, Leukoagglutinating phytohemagglutinin; MAA, *Maackia amurensis* agglutinin; NPL, *Narcissus pseudonarcissus* lectin; PSA, *Pisum sativum* agglutinin; SBA, Soybean agglutinin; SNA, *Sambucus nigra* agglutinin; STL, *Solanum tuberosum* lectin; UEA, *Ulex europeus* agglutinin-I; WFA, *Wisteria floribunda* agglutinin; WGA, Wheat germ agglutinin) were coupled to MyOne tosyl-activated magnetic Dynabeads from Thermo Fischer Scientific (Waltham, MA, USA).

Ten picomole (pmol) of the internal standard protein, chicken ovalbumin, was spiked into 50 µg of serum protein per pulldown. The sample was denatured in 20 mM Tris-HCl (pH 7.4), 20 mM dithiothreitol (DTT), 1% SDS, and 5% Triton X-100 for 30 min at 60 °C and alkylated in 100 mM iodoacetamide for 2 h in the dark at room temperature. Denatured serum was diluted 10-fold in binding buffer according to the following recipes: (i) Binding buffer for lectins EPHA, SNA, and STL (Binding buffer A)—20 mM Tris-HCl (pH 7.4), 0.2% SDS, 1 mM DTT, 150 mM NaCl, 1 mM CaCl2, 1 mM MnCl2, 1% Triton. (ii) Binding buffer for other lectins (Binding buffer B)—20 mM Tris-HCl (pH 7.4), 0.05% SDS, 1 mM DTT, 300 mM NaCl, 1 mM CaCl2, 1 mM MnCl2, 1% TritonL.

LeMBA-pulldown and tryptic digest was conducted on the Bravo liquid handler (Agilent Technologies, Santa Clara, CA, USA). After beads were aliquoted into wells, diluted serum samples were aliquoted into each well and then incubated with shaking for 1 h at 4 °C. After the incubation, the beads were washed 3 times in the same binding buffer and after the last wash, the beads were transferred into a new plate. The beads were then washed three times in 50 mM NH4HCO3 and transferred to a new plate again after the last wash. The beads were washed twice in 50 mM NH4HCO3 and transferred to a new plate for the last time after the last wash. The beads were washed once after the last transfer. To each well, 0.95 µg of modified sequencing grade trypsin (Promega. Madison, WI, USA) diluted in 20 µL of 50 mM ammonium bicarbonate was added and incubated in a shaking oven overnight at 37 °C. The supernatant was collected in a new plate and the beads were washed with 20 µL of 50 mM ammonium bicarbonate and mixed in the new plate. The supernatant was left to sit on the magnet to eliminate any carryover of beads and was transferred to a new plate. This process was repeated twice. Lastly, 20 µL of 5% formic acid was mixed with the supernatant and dried in a vacuum evaporator. The plates were kept at −80 until mass spectrometry analysis.

Tryptic peptides were subjected to LC-MS/MS using an Agilent 6520 QTOF (Santa Clara, CA, USA) coupled with a Chip CUBE and 1200 HPLC. The analytical or nano pump was set at 0.3 µL/min and the capillary pump at 4 µL/min. The HPLC-chip used contained a 160 nl trapping column, and a 150 mm, 300A, 5 µm C18 resolving column (Large Capacity Chip (II), Agilent Technologies, Santa Clara, CA, USA). For peptides from glycoprotein capture, the gradient went from 6% to 46% Buffer B (90% isocratic grade or gradient grade acetonitrile, 0.1% mass spectrometry grade formic acid in MilliQ water) in 40 min, 50% to 95% Buffer B at 41 min, plateaued at 95% until 56 min, and then returned to 6% Buffer B at 57 min until 67 min. The mass spectrometer was programmed to acquire 8 precursor MS1 spectra per second and 4 MS/MS spectra per second. Dynamic exclusion was applied after 2 MS/MS within 0.25 min.

The raw data files were processed with Spectrum Mill (Rev B.04.00.127, Agilent Technologies, Santa Clara, CA, USA). Data were extracted with carbamidomethylated cysteine as a fixed modification and then searched against an in-house canine database (described below). The following parameters were used for the search: 2 maximum missed cleavages, precursor mass tolerance of +/−20 ppm, and product mass tolerance of +/−50 ppm. For the search, carbamidomethylation was selected as the fixed modification and oxidized methionine was selected as the variable modification. Results were filtered by protein score >15, peptide score >6, and % scored peak intensity (% SPI, >60). Automatic validation was used to validate proteins and peptides with default settings, and false discovery rates (FDRs) were calculated using reversed database scores with the maximum peptide FDR threshold set to 1%. Only single peptide proteins with peptide score of >15, % SPI > 80, and up to 1 miss cleavage were included in the analysis.

Protein databases were downloaded from UniProt (SwissProt and TrEMBL; https://www.uniprot.org/; accessed on 9 January 2013), NCBI (https://www.ncbi.nlm.nih.gov/protein/; accessed on 4 February 2013) and Ensembl (https://www.ensembl.org/index.html?redirect=no; accessed on 23 February 2013) using *Canis familiaris* (Taxon: 9615) as the species. Sequences shorter than 30 amino acids were removed. The canine databases were merged together and reciprocal best hit (RBH) basic local alignment search tool (BLAST) was performed [14]. RBH BLAST, a BLAST method where the input and database file used are the same, was performed on the University of Queensland High Performance Computing Server to find more confident hits. Each match was given a score based on % identity, e-value, and coverage where the match was assigned +2 and +1 for a % identity of above 97.5% and 95%, respectively, +2 and +1 for an e-value of below 1E-180 and 1E-150, respectively, and +1 if coverage was above 0.95, resulting in scores ranging from 0 to 5. Priority was given to longer sequences and if the same length and similarity, in the following order: SwissProt, Ensembl, NCBI, TrEMBL, and GENESCAN. A total of 50,349 proteins remained which was used for subsequent BLAST, InterProScan, and mass spectrometry analysis.

Candidate selection was conducted using both sparse partial least squares-discriminant analysis (sPLS-DA) and group-binding difference via an in-house tool, GlycoSelector platform as previously reported [15]. Briefly, the intensities of ovalbumin peptides were extracted from Spectrum Mill and used for normalization. For each sample, all ovalbumin peptide intensities for all lectins were compiled into a single comma-separated value (csv) file and uploaded into GlycoSelector. Normalization of each peptide and calculation of the normalization factor was performed within GlycoSelector. Since each lectin binds to ovalbumin with different affinity, normalization was performed for each lectin across all samples. The normalization factor was calculated by dividing individual peptide intensities by the mean peptide intensity of all samples for that particular peptide for each lectin. Then, the mean of 4 or more ovalbumin peptides was calculated as the normalization factor.

The exported search file and internal standard file containing information for ovalbumin intensities for each run, along with information on the patient and sample, were uploaded into GlycoSelector. The following steps were taken in order: (1) sample outlier detection, (2) classification error, sensitivity, and specificity calculation, (3) stability analysis, and (4) sPLS-DA analysis. The top 100 lectin-protein candidates were exported, and those with stability scores higher than 0.6 were selected, resulting in 82 glycoproteins candidates determined by lectin-binding and protein accession (Table S1B).

In addition to sPLS-DA, group binding difference was also performed in GlycoSelector to identify biomarkers that were bound in more than 60% of one sample group but less than 40% of the other group and vice versa. Such flexible measures were used to identify as many candidates through this method as possible. Based on this criterion, group binding difference identified a total of 64 glycoprotein candidates with potential present/absent glycosylation changes (Table S1C). Combining with the sPS-DA-derived candidates, there were 49 individual proteins, with diverse lectin-binding glycoforms.

### 2.3. LeMBA-Multiple Reaction Monitoring-Mass Spectrometry (MRM-MS) for Biomarker Qualification

A custom-scheduled MRM method was developed for 58 proteins, 49 being discovery proteomics-derived candidates plus 8 literature-derived candidate proteins (angiopoietin-1, angiopoietin-4, vascular endothelial growth factor receptor 1, angiopoietin-1 receptor, vascular endothelial growth factor C, integrin alpha-V, ephrin type-B receptor 3, ephrin type-A receptor 1), and the internal standard protein chicken ovalbumin. The MRM Selector function of Spectrum Mill (Agilent Technologies, Santa Clara, CA, USA) was utilized to export potential transitions based on previous QTOF runs. The retention time and collision energy were optimized. For each protein, the number of peptides was limited to five with a score of above 10 and % score peak intensity of 70%. Only the y ions and the top five transitions > precursor m/z were selected. A maximum of 200 transitions was used to generate a method with a dwell time of 1 sec. The collision energy (CE) for each precursor was calculated using the formula: CE = 0.036 m/z−4.8. For both loading and analytical pumps, 3% Buffer B was maintained as baseline. The gradient run of the analytical pump was as follows: 10% B at 0.5 min, 40% B at 20.5 min, 95% B at 21.5 min plateauing until 23.5 min and dropping back down to 3% at 24.5 min until 27 min when the run was completed. The HPLC-chip used contained a 360 nl trapping column and a 150 mm, 180A, 3 µm C18 resolving column (Polaris-HR-Chip 3C18; Agilent Technologies, Santa Clara, CA, USA). Transitions without prominent peaks and shifting retention time were discarded.

The final method monitored a total of 362 transitions with an average of 3 peptides per protein and two products per peptide. The dwell time varied, depending on the number of transitions being analyzed, from 5.29 to 248.86 ms. For serum glycoprotein biomarker validation, LeMBA was performed using seven shortlisted lectins (AAL, DSA, LPHA, NPL, PSA, SNA, and WGA), chosen based on the number of candidates identified for the lectin, and the overall or mean ranking of candidates and then analyzed by the custom MRM assay. A total of N = 98 samples were analyzed (N = 30 from clinically normal dogs, N = 55 from HSA group and N = 33 from HSA-like cases (Table S2A)).

The resulting LeMBA-MRM-MS dataset (Table S2B) was filtered based on the following parameters (Table S3A–C): (i) peptides with median intensity of less than 500 across all samples were removed, (ii) peptides that were not detected in at least 90% of the samples in any one of the groups were removed, (iii) peptide intensity was Log2 transformed and median intensity (called dilution factor) of all the peptides for each sample was calculated. Samples showing median peptide intensity of Log2 > 1 or Log2 < 1 were removed. (iv) Correlation between all the peptides belonging to the same protein was performed and peptides with <0.6 Pearson correlation cut-off were removed. (v) Missing values were imputed as 1 (Log2(1) = 0). (vi) Samples were normalized based on the dilution factor. (vii) Peptide intensity was converted to protein intensity by giving equal weight to each peptide. Log2 (fold change) and *p*-value were calculated for each lectin-glycoprotein biomarker candidate. Data analysis was carried out using an in-house R script.

### 2.4. Samples for Lectin and Immunohistochemistry

Formalin-fixed paraffin-embedded (FFPE) and fresh-frozen splenic tumor tissues were collected between 2010 and 2017 from the Veterinary Medical Centre, the University of Queensland, and Brisbane Veterinary Specialist Centre (BVSC). Archival samples were provided by the Small Animal Specialist Hospital (SASH), Sydney. The sample collection at the University of Queensland Veterinary Medical Centre and BVSC was performed with University of Queensland Animal Ethics Committee approval (permit no. SVS/438/15/KIBBLE/CRF) and with the animal owner’s consent. The inclusion criteria prioritized cases presenting with an abnormal mass in spleen regardless of the final diagnoses. Cases with an HSA-suspected mass in other internal organs, such as liver and heart, were also collected.

Fifty-eight FFPE samples were selected after histopathologic diagnosis was made by pathologists (Table S4). Forty-nine samples were collected from spleen, six from liver, and one sample from heart, peritoneal mass, and sternal mass, respectively. Immunohistochemistry (see later) targeting CD31 and myeloid lineage antigen (MAC387) were performed on ambiguous samples to confirm the endothelial or macrophage/monocyte cell origin, respectively.

The samples were assigned to two groups, HSA (n = 32) and non-HSA (n = 26). For HSA samples, histopathological grades were applied based on (i) cancer differentiation, from well-differentiated (grade 1), moderately differentiated (grade 2) to poorly differentiated (grade 3), and (ii) growth patterns (typical slit-like, solid, cavernous, and mixed pattern) [16,17,18,19]. For the non-HSA group, the 26 samples were diagnosed as the following: adenocarcinoma (n = 1), apocrine gland tumor (n = 1), fibrohistocytic nodule (n = 1), hemorrhage (n = 2), hepatocellular carcinoma (n = 5), hematoma (n = 5), lymphoid hyperplasia (n = 1), lymphoma (n = 3), nodular hyperplasia (n = 1), normal spleen tissue (n = 3), splenitis (n = 1), ambiguous diagnosis (n = 2). One patient had two lesions presented, HSA and lymphoid hyperplasia. A summary of subject profiles is presented in Table S4.

### 2.5. Lectin-Histochemistry (LHC)

Five micrometer FFPE tissue sections were cut and placed on Menzel-Glaser Superfrost PLUS^®^ 25 × 75 × 1 mm^3^ microscope slides (Thermo^®^ Scientific, Waltham, MA, USA), heat-fixed for 90 min at 60 °C, then cooled before deparaffinization and rehydration. No antigen retrieval was performed prior to DSA and WGA histochemistry, while Heat-Induced Epitope Retrieval (HIER) using Tris/EDTA buffer, pH 9 (Dako^®^, Agilent Technologies, Santa Clara, CA, USA) was performed for SNA lectin. Blocking of nonspecific peroxidase activity and protein binding were performed by incubating with peroxidase (Peroxidase Block, Dako^®^, Agilent Technologies, Santa Clara, CA, USA) for 15 min at room temperature followed by 15 min incubation with 0.15 M glycine in PBS, where a quick rinse with Tris-buffered saline and Tween-20 (TBST) wash buffer (0.05 mol/L Tris-HCl, 0.3 mol/L NaCl, 0.1% Tween 20, 0.01%, pH 7.6; Dako^®^, Agilent Technologies, Santa Clara, CA, USA) was applied in between. This was followed by incubation with “antibody diluent with background reducing components ready-to-use” (Dako^®^, Agilent Technologies, Santa Clara, CA, USA) for 30 min at room temperature.

Glycan (sugar) chains of the tissue glycoproteins (GlcNAc)2-4, GlcNAc, Neu5Acα6Gal/GalNAc, and αMan/αGlc were detected by biotinylated lectins; DSA (*Datura stramonium*), WGA (wheat germ agglutinin), SNA (*Sambucus nigra*), and PSA (*Pisum sativum*), respectively (all from Vector Laboratories Ltd., Burlingame, CA, USA). Lectins and preferred sugar specificity are shown in Table S5. The slides were incubated in a moist chamber at room temperature for one hour. The binding was visualized using the avidin-biotin peroxidase complex method (Vectastain Elite^®^ ABC Kit Standard, Vector Laboratories Ltd., Burlingame, CA, USA) and the chromogen substrate 3-amino-9-ethylcarbazole (AEC), resulting in a bright red signal on a background of hematoxylin counterstain (Mayer’s Hematoxylin (Lillie’s Modification) Histological Staining Reagent; Dako^®^ Agilent Technologies, Santa Clara CA, USA). A summary of the lectin-histochemistry protocols is shown in Table S5. The sections, mounted with aqueous base mounting medium, Faramount^®^ (Dako^®^, Agilent Technologies), were examined by light microscopy and scanned (Olympus slide scanner VS120, Olympus^®^, Tokyo, Japan) for further analysis.

### 2.6. Immunohistochemistry (IHC)

For comparison to lectin-histochemistry results, immunolabeling of candidate glycoproteins, maltase-glucoamylase (MGAM), complement C7, and vitronectin (VTN) was performed, using anti-MGAM (HPA002270, Sigma Life Science, Saint Louis, MO, USA), anti-complement C7 (ab192346, abcam, Cambridge, UK), and anti-VTN (PA5-27909, Thermo Fisher Scientific, Waltham, MA, USA) antibody, respectively. A routinely used endothelial cell marker, CD31 (mouse monoclonal antibody Clone JC70A, M0823; Dako^®^, Agilent Technologies, Santa Clara, CA, USA), was used to confirm the endothelial cell origin of target cells. Mouse monoclonal anti-human myeloid lineage marker MAC 387 (Dako^®^, Agilent Technologies, Santa Clara, CA, USA) was used to aid in the diagnosis of ambiguous tissue samples, notable differentiation of has, and histiocytic proliferative diseases (in older studies in the literature known as “fibrous histiocytoma” [19]).

Following tissue deparaffinization and rehydration, antigen retrieval and blocking of non-specific binding sites were performed as described for lectin-histochemistry. Antigen retrieval methods and antibody dilutions for each of the antibodies are shown in Table S6. Sections were incubated with the primary antibodies in a moist chamber overnight at 4 °C. Detection of bound antibodies was performed using the Dako Envision system (EnVision+ System HRP; Dako^®^, Agilent Technologies, Santa Clara, CA, USA) with visualization using AEC substrate and counterstaining with Meyer’s hematoxylin.

To establish optimal dilutions for lectins and primary antibodies, various tissue antigen retrieval (AR) techniques including no-AR, HIER with Tris/EDTA buffer, pH9, citrate buffer, pH6 (Target Retrieval Solution, S2367, S1699, Dako^®^, Agilent Technologies, Santa Clara, CA, USA), and proteinase K treatment (Proteinase K Ready-to-use; Dako^®^, Agilent Technologies, Santa Clara, CA, USA) were trialed on two randomly selected HSA tissue samples. The optimized antigen retrieval techniques and dilutions for each ligand are shown in Table S5.

### 2.7. Positive and Negative Controls

To ensure constant signal intensity of the lectin/immunohistochemical labelling and to facilitate relative signal intensity scoring, a control for lectin/immunohistochemistry was chosen from trial experiments amongst several tissue blocks that had indisputable typical HSA features. Out of the sacrifice tissue block, two microscopic slides with 5 µm consecutive tissue sections were included in every experimental batch, one being a positive control and the other for a negative control. The internal positive controls were normal vascular endothelial cells in the samples, which showed lectin/immunohistochemical reaction at various intensities (see Results). For the CD31 ligand, archival baboon (*Papio* sp.) intestinal tissue (courtesy of Dr. P. Farrell, Univ. of Sydney, with approval from the SSWAHS Animal Welfare Committee) was used as positive control in addition to canine splenic tissue. For the external negative controls, antibody diluent was applied instead of the primary antibody/lectin, while the secondary antibody and chromogen were applied as described above.

### 2.8. Semi-Quantitative Lectin/Immunohistochemistry Scoring

To quantify the candidate glycoprotein expressions in HSA tissues compared to non-HSA, each sample was assessed by components that expressed the glycoproteins, i.e., (i) HSA cells, (ii) arterial endothelial, and (iii) venous endothelial cells. The scoring system used for the assessment of lectin/immunohistochemical reaction in this study was modified from the H-score system, which is widely used for evaluating immunohistochemical reaction signal intensity expressed by tissues/cells such as for the evaluation of steroid receptors in breast cancer [20]. The scores were given subjectively to each tissue component separately.

The data collected were based on the assessment of two parameters for tissue glycoprotein expression: the intensity level of the lectin/immunohistochemical reaction (mild, moderate, high or 1–3) and the proportion of cells expressing the glycoprotein as a percentage. When combining those two parameters, the H-scores ranged between 0 and 300, which then were classified into four overall intensities from no signal detected to high signal intensity (Figure S1).

### 2.9. Statistical Analysis

The overall intensities of the lectin-immunohistochemical signal of each ligand and tissue components of the samples were assessed. The comparisons were performed at three levels. The first level, microscopic components, was to compare lectin-immunohistochemical signal intensities expressed by each antibody/lectin across tissue components, i.e., HSA cells, arterial endothelium, and venous endothelium, as described in the previous section. The Wilcoxon-signed rank test was applied to test for the difference between each pair of tissue components. Bonferroni correction was performed to adjust *p*-values for multiple comparison. The second level was to compare the signal intensities across two sample populations: dogs with HSA and dogs without HSA. The Kruskal–Wallis test was applied for this comparison. The graphical illustrations and statistical analysis were created and performed using R (3.5.1–3.6.1) and R package, ggplot2 [21].

The third level was to assess the performance of those lectins/antibodies used in the study, by prioritizing the reagents that had the ability to distinguish between HSA tissues and non-HSA tissues. In this analysis, the LHC/IHC H-scores of “HSA” tissue samples (n = 33) were compared with tissue samples that had similar histologic morphology to HSA, but were not HSA, i.e., “HSA-like” (n = 6). HSA-like samples included four splenic hematomas and two cases of hemorrhage. Data were visualized by matrix scatter plots and analyzed by Recursive Partitioning using R package, Rpart [22].

In addition, the trends of relationship between lectin/immunohistochemical signal intensity by H-scores and HSA histopathological grades or growth pattern were also assessed. For the relationship between H-scores and HSA histopathological grades, the Jonckheere–Terpstra trend test and testing of linear model fitting were performed using clinfun, R package [23]. The Kruskal–Wallis test was applied for assessing the difference of H-scores among HSA growth patterns. For all statistical analyses, *p*-values < 0.05 were considered statistically significant.

## 3. Results

### 3.1. Serum Glycoprotein Biomarker Candidates

**Figure 1:**
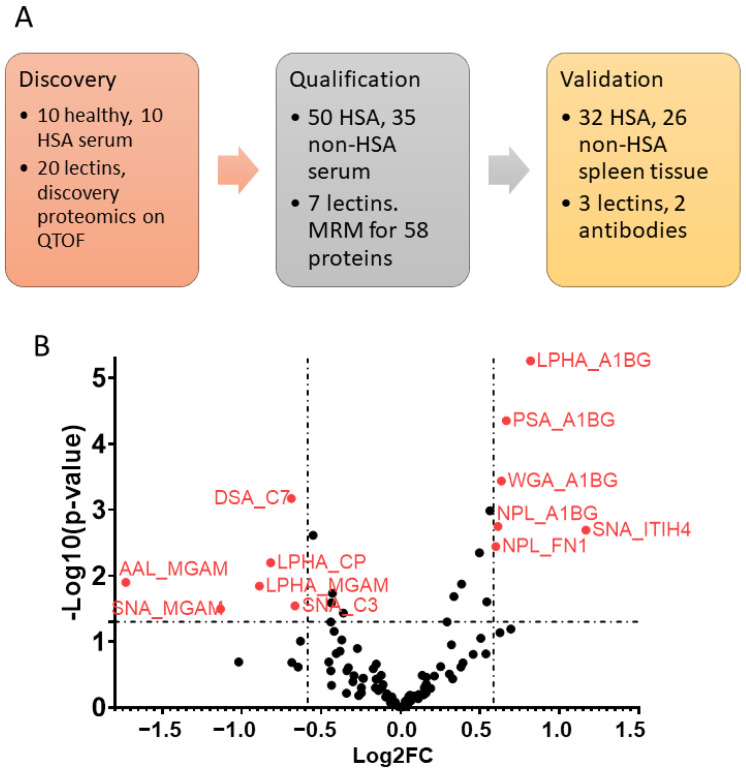
Serum biomarker discovery and validation workflow for canine hemangiosarcoma (HSA). (**A**) Sample numbers and approaches for each biomarker phase. (**B**) Volcano plot of serum biomarker candidate validation by LeMBA-MRM-MS. The candidates with 1.5-fold change and *p*-value < 0.05 are highlighted.

The biomarker discovery workflow is depicted in Figure 1A. From the validation phase data, setting 1.5-fold change as a threshold and *p*-value < 0.05 as cut-off, six candidates were upregulated and six candidates were downregulated in HSA compared to non-HSA (Figure 1B). The upregulated glycoprotein candidates included four different lectin binding forms of α-1B-glycoprotein (A1BG), NPL-binding fibronectin 1 (FN1), and SNA-binding Inter-Alpha-Trypsin Inhibitor Heavy Chain 4 (ITIH4). The glycoproteins found to be downregulated in sera from dogs with HSA compared to non-HSA were three different lectin binding forms of maltase-glucoamylase (MGAM), DSA-binding complement component 7 (C7), SNA-binding complement component 3 (C3), and LPHA-binding ceruloplasmin (CP).

### 3.2. Establishing Control Framework for LHC/IHC Analysis

In order to ascertain whether glycoproteins detected by LeMBA-MRM-MS in serum from dogs with HSA originated from the neoplastic endothelial cells or from adjacent tissues reacting to the presence and growth of the neoplastic cells, we applied LHC and IHC to tissues with HSA and other neoplasms, diagnosed by histopathology by veterinary pathologists, as well as to non-cancerous tissues.

**Figure 2:**
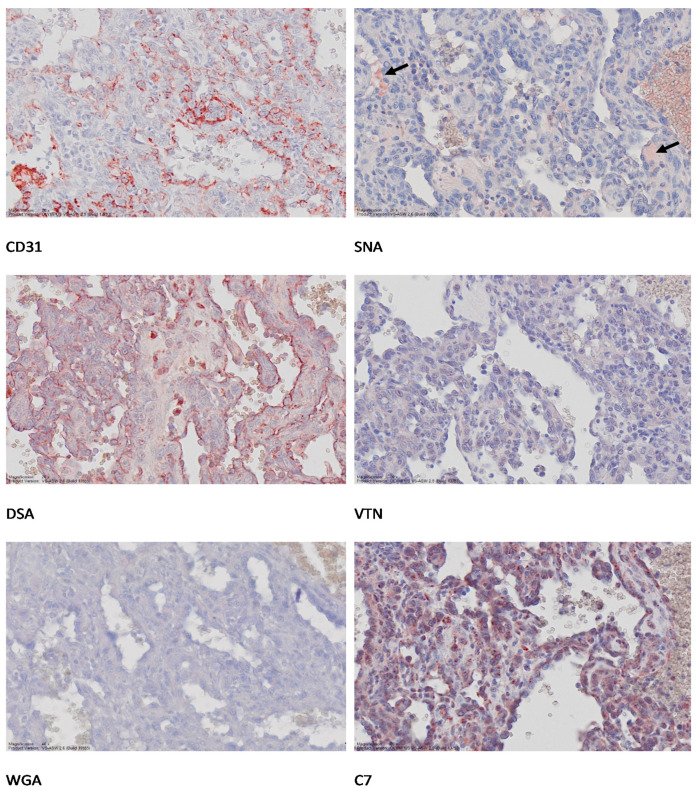
Lectin-histochemistry and immunohistochemical labelling of canine splenic HSA. Formalin-fixed paraffin-embedded (FFPE) sections were reacted with anti-human CD31 antibody, anti-human VTN antibody, anti-human complement C7 antibody, and biotinylated lectins DSA (*Datura stramonium*), WGA (Wheat Germ Agglutinin), and SNA (*Sambucus nigra*) lectins) on consecutive splenic HSA sections (sample 13_019400E). Binding was visualized using AEC substrate (red) following either incubation with secondary antibody (Envision Kit) or streptavidin-horse radish peroxidase conjugate. Microphotos were generated using Aperio scans. Original magnifications 20×.

Even though the tissue trimming process aimed to collect tissue samples where the normal/cancer tissue margin was presented, not all tissue sections contained every component of interest (cancer cells, endothelium of normal arteries, and veins). Poor quality tissue samples (e.g., extensive tissue necrosis, containing more than 50% area of cell debris and/or blood clots) were excluded from the analysis. Consecutive tissue sections from the selected control HSA sample (sample with indisputable HSA characteristics—refer to Figure 2) were included in every batch of lectin-immunohistochemistry labeling. A summary of the number of experimental repeats, average H-scores for each ligand, and the Coefficient of Variations (CV, %) for the control sample is presented in Table S6.

CD31 immunolabeling was used to confirm the endothelial cell origin of the HSA tissue samples. The signal for CD31 immunohistochemical labeling on vascular endothelium appeared bright red when visualized with AEC (Figure 2, Figures S2 and S3). Notably, in canine spleen with HSA, CD31 mainly labelled arterial endothelium rather than venous endothelium, as indicated by the overall H-score (Tables S7 and S8). In contrast, in baboon tissue, the CD31 immunohistochemical signal intensities were almost similar for arterial and venous endothelium (average H-score = 280.8 for arterial endothelium and 275.4 for venous endothelium; Table S6).

### 3.3. Lectin Histochemistry

DSA: Thirty-two HSA tissue samples were included in the DSA histochemistry. All samples expressed DSA-bound glycoproteins with various intensities. Among the three target tissue components, HSA cells, arterial endothelium, and venous endothelium, a DSA histochemical signal was observed with the highest intensities in the cytoplasm of venous endothelium compared to arterial endothelium, where heterogeneous signal intensities were observed in HSA cells (Tables S7–S9). Examples of labelling are shown in Figure 2, Figures S2 and S3. Labelling was also observed on some mononuclear cells, red blood cells, and cellular debris, the latter considered a non-specific reaction.

Blocking of the DSA lectin reactions with a specific blocking agent, chitin hydrolysate (Vector^®^ Laboratories Ltd, Burlingame, CA, USA.), was tested in four different conditions: (i) prior to lectin incubation, the tissues were pre-incubated with 1:10 dilution of Chitin hydrolysate for 1 h at 37 °C; (ii) 1:5 dilution for 1.30 h at 37 °C; (iii) 1:5 dilution overnight at 4 °C followed by incubation with DSA; and (iv) using Chitin hydrolysate instead of antibody diluent for the DSA lectin solution. The best result was obtained with overnight incubation at 4 °C, but even that gave incomplete blocking of DSA binding sites.

WGA: WGA histochemistry also labelled the three tissue components. However, the overall H-scores observed for venous endothelium and arterial endothelium were the opposite of what was seen for DSA: less to no histochemical signal was observed on the venous endothelium, while most of the histochemical signal was observed in the arterial endothelium (Tables S7–S9; Figure 2, Figures S2 and S3). Labelling was observed on some of the mononuclear cells in the spleen. Background staining was common, especially on cellular debris.

SNA and VTN: Unlike WGA and DSA, the lectin histochemical signals expressed in the cell components were very low to undetectable. Low histochemical signal intensity was observed in the endothelial cells of the arteries, while almost none was observed in the endothelial cells of the veins. The HSA cells also expressed low lectin histochemical signal, while most of the positive lectin histochemical reaction was observed extracellularly, either in between chambers of the slit-like HSA tissues or in vascular lumens (Figure 2, Figures S2 and S3). The labelling pattern of VTN was similar to that of SNA, with signal intensity low and appearing extracellularly rather than intracellularly, as shown in Figure 2, Figures S2 and S3.

### 3.4. Immunohistochemistry for Complement C7

The immunohistochemical signal for complement C7 was observed in the cytoplasm of HSA cells and appeared granular (Figure 2, Figures S2 and S3), with signal intensity being relatively uniform across each sample. Some labelling was also observed in mononuclear cells and macrophages in the spleen.

### 3.5. Glycoproteins Expression on the Tissue Components Assessed by H-Scores

**Figure 3:**
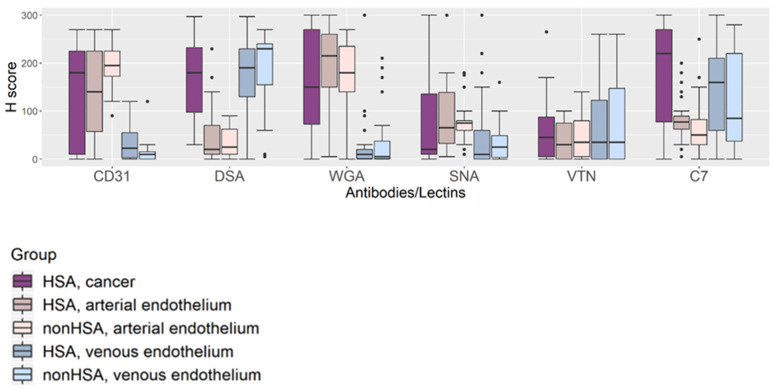
Boxplot representing overall lectin-histochemistry (DSA, WGA, SNA) and immunohistochemical (CD31, VTN, C7) signal intensities. Three tissue components—HSA/cancer, arterial endothelium, and venous endothelium—were assessed in two sample groups—dogs with HSA and dogs without HSA. Differences in the level of tissue glycoprotein expression between tissue components and between HSA and non-HSA were compared.

Overall, H-scores assessed in the HSA cells, arterial endothelium, and venous endothelium were compared and statistically analyzed. The results demonstrated varying levels of lectin and immunohistochemical signal, representing tissue glycan or glycoprotein expression (Figure 3). The actual distribution of the H-score assessed in HSA cells (cancer component), arterial endothelium, and venous endothelium are displayed in Figure S4.

### 3.6. Comparison of the Tissue Glycoprotein Expression of Normal Vascular Endothelium between HSA Tissues and Non-HSA Tissues

**Figure 4:**
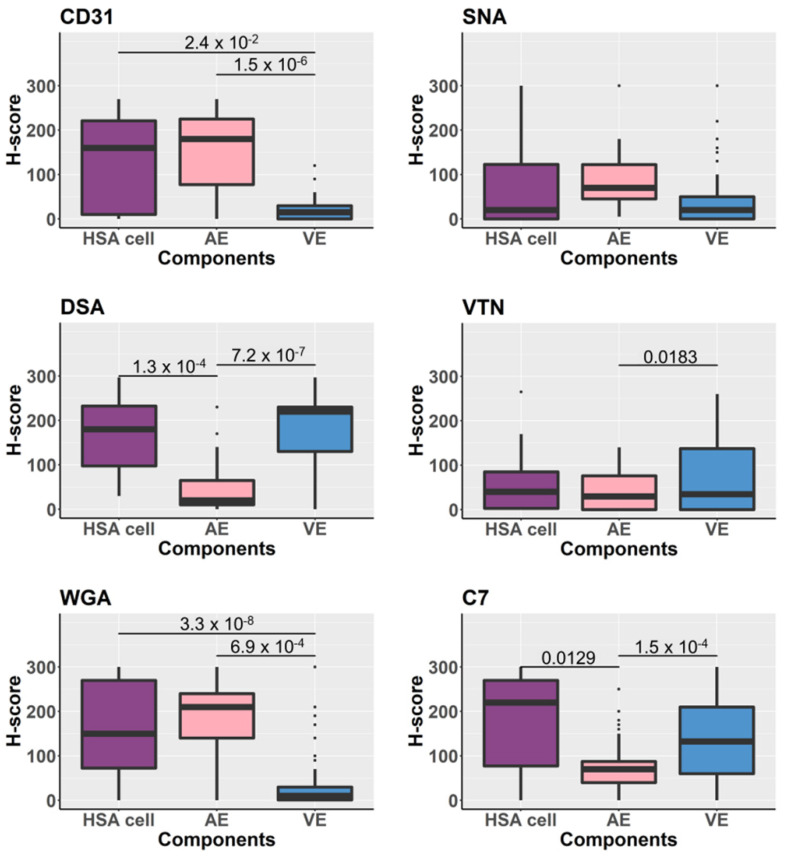
Boxplots demonstrate the comparison of H-scores assessed from lectin-histochemistry (DSA, WGA, SNA) and immunohistochemical (CD31, VTN, C7) labelling of tissue samples. The statistical analysis was performed by comparing H-scores between tissue components—HSA/cancer cells, arterial endothelium (AE) and venous endothelium (VE). Significance bars show only *p*-values < 0.05 derived from Wilcoxon test and adjusted by Bonferroni corrections.

The results described above showed that the expression of glycoproteins differed for arterial and venous endothelial cells, respectively, as demonstrated by the overall H-scores for lectin-histochemistry and immunohistochemical labelling. We, therefore, compared overall H-scores of the arterial and venous endothelium among two disease condition groups: tissue samples from dogs with HSA (HSA) and from dogs without HSA (non-HSA). The boxplots in Figure 4 demonstrate the comparison for each ligand. There was no statistically significant difference among the glycoprotein expressed by either arterial or venous endothelial cells. The results suggested that glycoproteins or glycans expressed by normal vascular endothelium of the spleen were not altered by the presence of HSA in the organ. This supports the use of adjacent normal vascular endothelium as an internal control when performing lectin/immunohistochemistry using the lectins and antibodies employed in this study.

### 3.7. Associations between HSA Grades and Growth Patterns and Expression of Glycans/Glycoproteins Detected by Lectin-Histochemistry and Immunohistochemistry

The histopathological grades were applied to thirty-two HSA samples based on cancer differentiation, from well-differentiated (grade 1), moderately differentiated (grade 2) to poorly differentiated (grade 3) [17,18,19]. The majority of the HSA samples (n = 14) were moderately differentiated, while fewer samples (n = 11) were poorly differentiated and only seven samples were well-differentiated HSA. The growth patterns of the HSA were also identified as: typical (n = 10), solid (n = 7), cavernous (n = 2), and mixed pattern (n = 13).

For CD31, which was considered the standard marker of endothelial cell origin, the HSA cells of the well-differentiated and poorly differentiated cancers tended to express lower levels of CD31 than those of the moderately differentiated HSA. No statistically significant differences among histopathological grades were observed after testing by the Jonckheere–Terpstra trend test, with the obtained *p*-values = 0.62. However, the dispersion of H-scores, demonstrated by the length of the boxplot (Figure S5) and the IQRs (grade 1 = 10, grade 2 = 85, and grade 3 = 192.5; Table S9), increases along with the cancer grades.

Levels of DSA histochemical reaction on HSA cells and venous endothelial cells assessed by H-score were statistically significantly different among histopathological grades, with increasing DSA-expression as the HSA became less differentiated (Figure S5). The Jonckheere–Terpstra trend test returned *p*-values = 0.04 and the fitting of linear model testing yielded a *p*-value = 0.026 for the F-statistic.

For SNA lectin, there was a borderline trend of decreasing HSA cell expression of the SNA-binding glycoproteins with decreasing differentiation, when performing the Jonckheere–Terpstra trend test and fitting of linear model testing, with *p*-values = 0.07 and 0.06, respectively. No significant differences in lectin/histochemical reaction among three HSA histopathological grades were observed for WGA, VTN, and C7 lectin/histochemistry (Figure S5).

For growth patterns assessment, there was only a limited number of samples with cavernous growth pattern. Hence, only solid and typical types of HSA growth patterns were included for the comparison. No statistical differences between the overall H-scores were detected. However, for the solid type of has, there was a trend of lesser expression of CD31, complement C7, VTN, DSA, and SNA bound glycoproteins than in typical HSA (Figure S6). In contrast, WGA bound glycoproteins gave the opposite result: solid HSA was more likely to express higher WGA bound glycoproteins than typical HSA (Figure S6).

### 3.8. Potential HSA Tissue Markers Assessed by Recursive Partitioning

The final objective was to select top reactants among the six antibodies/lectins (as tissue markers) that could best distinguish HSA from HSA-like according to their differences in the level of lectin-immunohistochemical signal intensities. The expected patterns of signal intensities were either high in HSA/low in HSA-like or vice versa.

**Figure 5:**
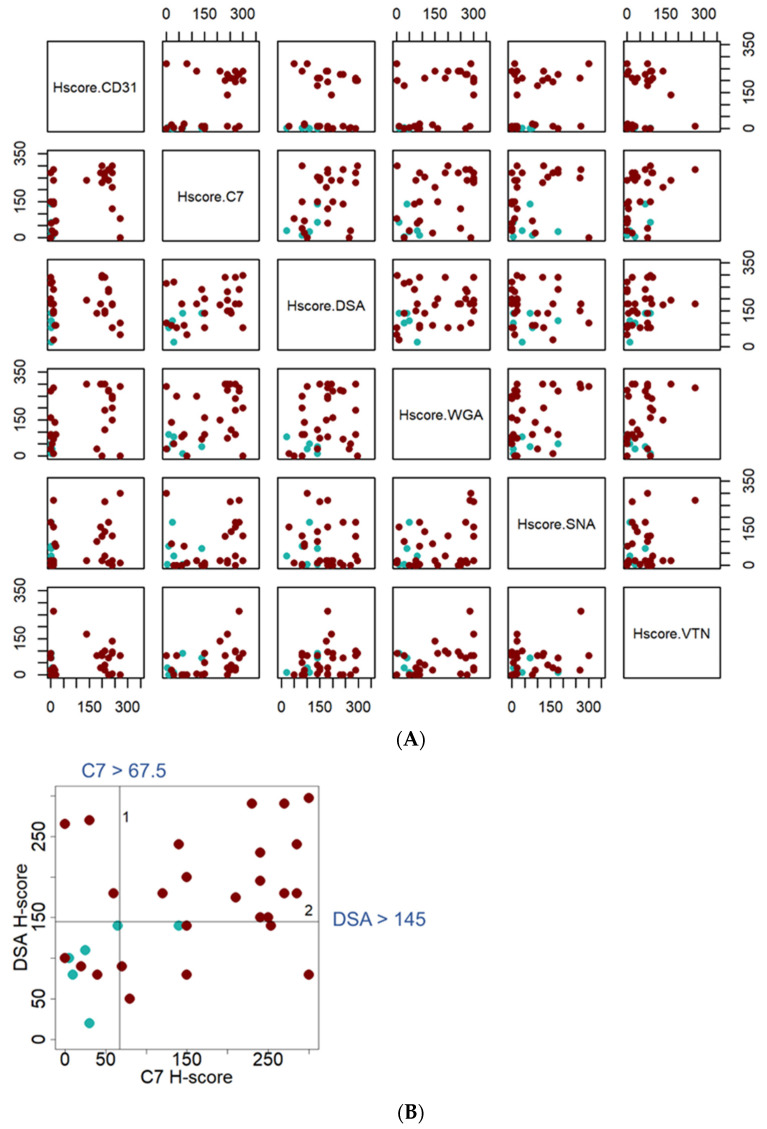
Scatter plots of H-scores distribution, assessed from lectin-immunohistochemical labelling for complement C7 and VTN as well as DSA, WGA, and SNA. (**A**) Matrix scatter plots created for the candidate antibodies/lectins H-scores. The darker dots (dark brown) represent H-score assessed from the cancer component of HSA splenic tissue samples, while the lighter dots (light green) represent H-score assessed from the HSA-like lesion of non-HSA (hematoma and hemorrhage) splenic tissue samples. (**B**) Scatter plot of H-scores distribution assessed from lectin-immunohistochemical labelling with the anti-human complement C7 antibody and DSA lectin. The pair of reactants was selected from recursive partitioning results. Two cut-off points, complement C7 (H-score > 67.5) then DSA (H-score > 145), allow clear distinction between HSA and HSA-like tissues.

A matrix scatter plot of the sample H-scores distribution when paired with different possibilities was created (Figure 5A). Recursive partitioning results suggested two of the markers tested would be useful in distinguishing between HSA and HSA-like tissues based on cut-off point values. The complement C7 antibody and DSA lectin were the top two target-reagents based on this set of data. Two cut-off points, at the C7 H-score above 67.5 and DSA H-score above 145, allowed for conclusive distinction of HSA tissue samples from HSA-like tissue, as shown in Figure 5B.

## 4. Discussion

Many serum cancer markers are glycoproteins, but discovery of new serum protein markers is highly challenging due to the complexity of the serum proteome. In this study, we applied a multi-phase biomarker discovery and validation glycoproteomics workflow and discovered novel serum glycoprotein biomarkers for canine HSA, followed by tissue immuno- and lectin histochemistry validation in independent samples. The highly interdisciplinary approach confirmed DSA and C7 staining as potential canine HSA diagnostic markers.

There were three reasons for manual LHC/IHC signal intensity assessment or a semi-quantitative method to be chosen over image analysis software or an immunofluorescence technique: (i) The organ of primary interest, the spleen, contains large quantities of red blood cells and background labelling “noise” was common. Immunofluorescence labelling was attempted but yielded no promising results. Eventually, bright red colored 3-amino-9-ethylcarbazole (AEC) substrate chromogen was used for lectin/histochemical reaction visualization instead of the commonly used 3,3′-diaminobenzidine, which presents in brown color and can mimic the color of erythrocytes in some samples and hemosiderin in splenic macrophages. (ii) There was no specific antibody or lectin which only labelled HSA cells without labelling the adjacent normal endothelial cells encountered elsewhere in the tissue samples. The image analysis software would need to be able to distinguish these cells based on morphology alone, which is still a challenging task, although machine learning algorithms may eventually be possible [24]. (iii) HSA is a non-uniform cancer; it has various growth patterns and degrees of differentiation [17,18,19]. Due to the nature of the samples, semi-quantitative LHC/IHC was considered the most suitable data collection method for this study design. It is acknowledged that the tissue pre-processing (sample collection, preservation, paraffin embedding) might contribute to inconsistencies of LHC/IHC reactions. However, considering that glycoprotein expression on normal vascular endothelium was consistent between samples, it may be concluded that sample handling and processing were of lesser concern.

Small sample size and non-comprehensive sampling are other common limitations when studying archival samples of naturally occurring diseases. The larger distribution of moderate and high-grade HSA in the sample population may, at least in part, reflect that the animals were diagnosed when the cancer had already advanced to a critical clinical stage, although it should be noted that there is no documented staging of HSA based on histopathology. An attempt to minimize the effect of this limitation was to apply two dimensions of data observation—the modified H-scoring system [20]. The samples are tissue sections which comprise >50 million cells per 5 × 10 mm^2^ tissue section, assuming the 5 µm-thick tissue section contains one cell layer. The size of the tissue sections included in this study varied between 5 × 10 mm^2^ to 20 × 30 mm^2^, equivalent to at least 2900 million cells. The H-scoring system allowed us to gather information from the LHC/IHC results at a deeper level, not only binomial, but the level of signal intensity plus proportion of cells in each level. It was shown to be a robust approach to protein expression measurement, which could be applied in automated quantitative immunohistochemistry for measuring human epidermal growth factor receptor 2 (HER2) expression in breast cancer [25]. Depending on the nature of samples, various IHC scoring systems may be assessed to determine the most suitable for the study [26]. For spleen samples, the H-scoring system appeared useful for LHC/IHC as the signal intensity of reactants could vary across the sample.

In addition to the HSA cancer cells, arterial and venous endothelial cells also showed heterogeneity of glycoprotein expression. Distinctive patterns of glycoprotein expression on different types of vascular endothelium (artery vs. vein) were demonstrated. Dog spleen arterial endothelium expressed greater levels of CD31 and WGA targets than venous endothelium, while greater expression of VTN, complement C7, and DSA targets was detected in the venous endothelium. This is in line with findings in humans, where arterial and venous endothelial cells expressed proteins differently and molecular identification markers for arterial and venous endothelium have been established: VEGF-R2, Notch, and Ephrin-B2 for arteries; BRG1, COUP-TFII, and Eph-B4 for veins (reviewed in [27,28]). Based on this finding, caution should be exercised when assessing the LHC/IHC signal intensity of endothelial cell markers on dog endothelial cells, i.e., the type of endothelial cell should be clearly identified and recorded.

The heterogeneity of LHC/IHC reactions on HSA cells may be due to the heterogenous nature of the cancer, where cell functions may have been altered and protein expression in the cells likewise changed over the course of development [29]. In contrast, there was consistency in the LHC/IHC signal intensity on arterial and venous endothelia, respectively, for all the antibodies and lectins used, regardless of whether the spleen contained HSA or not. Therefore, normal vascular endothelium can be used as an internal control, when performing LHC/IHC labelling with anti-human CD31, VTN, complement C7 antibodies, and DSA, WGA, and SNA lectins on dog spleen tissue. Whether this will apply to other endothelial molecules remains to be ascertained but seems likely.

No statistical difference was found when glycoprotein expression was compared between normal vascular endothelial cells of the HSA and non-HSA groups. This suggests that differences in the levels of serum glycoprotein biomarkers (Figure 1) were unlikely to be influenced by normal vascular endothelium, but rather that HSA cells or possibly liver, if the cancer has an effect on liver functions [30], were the source of such changes. Still, the roles of normal vascular endothelia in HSA cannot be completely ruled out based on this study alone, as there were two limitations to the study: the relatively small number of samples examined and the heterogeneity of the non-HSA group. The non-HSA group included various splenic lesions and only three out of 26 tissue samples were normal spleen. It is possible that other disease conditions could affect the level of glycoprotein expression of the vascular endothelium as well. To validate this, comparison of vascular endothelial cell glycoprotein expression between three groups—HSA, non-HSA, and healthy control in a larger cohort—will have to be conducted.

The trend in decreasing HSA cell expression of SNA bound glycans or glycoproteins as the cancer becomes more poorly differentiated, which is the opposite of what was seen for DSA bound glycans or glycoproteins, suggests SNA bound glycans may be involved in the HSA cancer biological process as well. However, the evidence from this study is not as robust as for the DSA bound glycans. Previously, glycosylation changes in HSA cancer cells were demonstrated by lectin immunohistochemistry using a panel of lectins, but DSA and SNA were not included [31]. The question remains what the underlying reasons for the altered lectin-histochemical signal intensities are, i.e., whether it is changes in the proteins or the glycan parts of the glycoproteins or both. To determine this will require that the DSA and SNA bound glycoproteins are identified, with complement C7 and C3, MGAM, and ITIH4 being amongst the likely candidates (Figure 1). However, there could be additional glycoproteins not detected in this study. Some of the possibilities of biological events leading to changes in glycoprotein expression in HSA are (i) impaired function of the cell protein synthesis process (impaired transcription, translation); (ii) protein is synthesized but cannot be conjugated with the glycans (post translational modification problems); or (iii) glycan changes (increase or decrease) by an unknown mechanism [32]. Thus, further studies with a focus on glycans are required.

Complement C7 is overexpressed in HSA cells compared to the immunohistochemical signal presented on hematoma tissues. This finding suggested complement C7 may be involved in HSA cancer biology. Complement C7 is a glycoprotein that forms a membrane attack complex (MAC) together with complement components C5b, C6, C8, and C9 [33]. The C7 gene is conserved in human and some other species, including dog. Their DNA and amino acid sequences are 84.4% and 81.4% identical, respectively [34]. Apart from the liver, complement C7 is also secreted by endothelial cells [35]. Proposed roles of complement C7 in ovarian and non-small cell lung cancer are inflammatory process regulation and possibly tumor suppression [36,37]. In hepatocellular carcinoma, complement C7 was highly expressed in the cancer cells, where the role was demonstrated to be maintenance of tumor initiating cell stemness [38]. Since common sites for canine HSA are spleen and liver, which both contain large numbers of immune cells, it could be surmised that modulation of inflammatory responses is a key function of complement C7 in this cancer and hence, the upregulation.

## 5. Conclusions

In conclusion, potential HSA biomarker candidates were identified and the locations of some of these candidates and the lectin-binding glycan/glycoprotein expression profile in spleen were demonstrated. Levels of glycoprotein expression were assessed using a semi-quantitative lectin/immunohistochemistry approach. The results suggest that complement C7 and DSA may supplement the use of CD31 and von Willebrand factor in IHC to confirm vascular endothelial cell origin in HSA suspected tumors. However, practicality should be evaluated in a larger sample size, especially with more and greater variety of controls. The results suggest that complement C7 may play a potential role in HSA biology. Identifying further glycoproteins which bind the lectins DSA and SNA may also be key to a better understanding of HSA pathobiology and should be pursued.

## Data Availability

Data generated in this project are stored in the University of Queensland Data Management System and may be made available upon request to M.M.H. and H.B.-O.

## Supplementary Materials

The following are available online at https://www.mdpi.com/2306-7381/8/3/38/s1. Table S1. (A) Case description used for glycoprotein biomarker discovery and (B) discovery phase candidates from sPLS-DA analysis, and (C) Candidates from GlycoSelect Group Binding Difference. Table S2. (A) Case description used for biomarker validation by lectin-coupled MRM assay, (B) proteins measured in the MRM method. Table S3. Filtering of the LeMBA-MRM-MS dataset. Table S4. Patients profile summary for samples used in lectin/immunohistochemistry. Table S5. Lectins and antibodies used in the study and their antigenic retrieval (AR) methods and dilutions. Table S6. Average H-scores for lectin/immunohistochemical signal intensities of each component of the control sample. Table S7. Results of lectin-histochemistry and immunohistochemical labeling of canine spleens with HSA. Table S8. Results of lectin-histochemistry and immunohistochemical labelling of canine non-HSA samples. Table S9. Overall, H-scores assessed from lectin/immunohistochemistry. Figure S1. Microscopic images demonstrating the visualization of the glycoprotein expression in the tissues. Figure S2. Comparison of labelling patterns for lectins (DSA, WGA, SNA) and anti-CD31, anti-C7 and anti-VTN antibodies on HSA tumor cells, arterial and venous endothelial cells. Figure S3. Examples of immunohistochemical and lectin-histochemistry labelling of canine splenic HSA with different growth patterns. Figure S4. H-score distribution of lectin- and immunohistochemical labelling of HSA cancer cells, arterial and venous endothelial cells. Figure S5. Boxplots of H-score distribution. Figure S6. Boxplots of H-score distribution, assessed for CD31, DSA, WGA, SNA, VTN and C7 lectin/immunohistochemical labelling signal intensity.
