# Supplementary material for: Candidate Glycoprotein Biomarkers for Canine Visceral Hemangiosarcoma and Validation Using Semi-Quantitative Lectin/Immunohistochemical Assays
Source: Vet Sci. 2021 Feb 27;8(3):38. doi: 10.3390/vetsci8030038 (PMC7997418; doi:10.3390/vetsci8030038)

**Supporting information & data.**

**Supplementary Table 1A. Case description used for glycoprotein biomarker discovery.**

| Group | Sample no. | Age | Sex | Breed |
| --- | --- | --- | --- | --- |
| Normal | 1 | 6 | M | Staffordshire Bull Terrier cross |
|  | 2 | 12 | MN | Border Collie cross |
|  | 3 | 8 | FS | Australian Cattle Dog |
|  | 4 | 8 | MN | Fox Terrier |
|  | 5 | 6 | FS | Rhodesian Ridgeback cross |
|  | 6 | 9 | MN | Labrador Retriever |
|  | 7 | 8 | MN | Labrador Retriever |
|  | 8 | 13 | FS | Labrador Retriever |
|  | 9 | 8 | MN | Border Collie |
|  | 10 | 9 | FS | Labrador Retriever |
| Average |  | 8.78 ± 2.38 |  |  |
| HSA | 1 | 8 | FS | Mixed |
|  | 2 | 10 | M | Australian Cattle Dog cross |
|  | 3 | 10 | MN | Australian Stumpy Tail Cattle Dog |
|  | 4 | 8 | MN | Miniature Schnauzer |
|  | 5 | 8 | MN | Scottish Terrier |
|  | 6 | 8 | MN | German Shepherd Dog |
|  | 7 | 7 | MN | Golden Retriever |
|  | 8 | 10 | FS | Labrador Retriever |
|  | 9 | 11 | FS | Labrador Retriever |
|  | 10 | 11 | MN | Border Collie |
| Average |  | 9.10 ± 1.45 |  |  |

**Supplementary Table 1B. Discovery phase candidates from sPLS-DA analysis.**

| **Rank** | **Candidate** | **Accession** | **Stability**  **score** | **Rank** | **Candidate** | **Accession** | **Stability**  **score** |
| --- | --- | --- | --- | --- | --- | --- | --- |
| 1 | WFA-C4A | F1PWR2 | 1 | 42 | NPL-GSN | E2QZK2 | 1 |
| 2 | LPHA-C4A | F1PWR2 | 1 | 43 | DSA-CFI | E2R7A3 | 1 |
| 3 | NPL-C4A | F1PWR2 | 1 | 44 | ConA-C3 | 73987236 | 1 |
| 4 | MAA-C4A | F1PWR2 | 1 | 45 | STL-ITIH4 | ENSCAFT00000039632 | 1 |
| 5 | WGA-CFI | E2R7A3 | 1 | 46 | NPL-RBP4 | 73998292 | 1 |
| 6 | SNA-LRG1 | E2R833 | 1 | 47 | JAC-ITIH1 | F1Q418 | 1 |
| 7 | DSA-C4A | F1PWR2 | 1 | 48 | HPA-C3 | 73987236 | 1 |
| 8 | PSA-C4A | F1PWR2 | 1 | 49 | EPHA-C4A | F1PWR2 | 1 |
| 9 | ConA-C4A | F1PWR2 | 1 | 50 | ECA-FN1 | 74005698 | 0.95 |
| 10 | PSA-CFI | E2R7A3 | 1 | 51 | SNA-CP | 73990371 | 0.95 |
| 11 | MAA-A1BG | 73947277 | 1 | 52 | GNL-ITIH4 | ENSCAFT00000039632 | 1 |
| 12 | ECA-C4A | F1PWR2 | 1 | 53 | AAL-ITIH2 | F1PG39 | 1 |
| 13 | SNA-SERPING1 | F1PYX9 | 1 | 54 | STL-PON1 | E2RPW3 | 0.95 |
| 14 | NPL-C5 | F1P7J4 | 1 | 55 | AAL-ITIH4 | ENSCAFT00000039632 | 1 |
| 15 | JAC-C4A | F1PWR2 | 1 | 56 | MAA-CD5L | F1PG16 | 0.89 |
| 16 | WFA-A1BG | 73947277 | 1 | 57 | PSA-FN1 | 74005698 | 0.89 |
| 17 | MAA-CFB | 73972310 | 1 | 58 | WFA-PLG | F1Q421 | 0.84 |
| 18 | DSA-C5 | F1P7J4 | 1 | 59 | LPHA-A1BG | 73947277 | 0.79 |
| 19 | SNA-C4A | F1PWR2 | 1 | 60 | EPHA-ITIH4 | ENSCAFT00000039632 | 1 |
| 20 | DSA-PON1 | E2RPW3 | 1 | 61 | GNL-CFI | E2R7A3 | 0.89 |
| 21 | STL-CFI | E2R7A3 | 1 | 62 | MAA-APOH | P33703 | 0.95 |
| 22 | NPL-CFH | E2RK21 | 1 | 63 | LPHA-PLG | F1Q421 | 0.79 |
| 23 | DSA-ITIH4 | ENSCAFT00000039632 | 1 | 64 | HAA-APOH | P33703 | 0.84 |
| 24 | JAC-GSN | E2QZK2 | 1 | 65 | AAL-CD5L | F1PG16 | 0.89 |
| 25 | EPHA-CFI | E2R7A3 | 1 | 66 | AAL-GSN | E2QZK2 | 0.79 |
| 26 | WGA-ITIH4 | ENSCAFT00000039632 | 1 | 67 | PSA-ITIH4 | ENSCAFT00000039632 | 0.95 |
| 27 | JAC-C5 | F1P7J4 | 1 | 68 | NPL-CFI | E2R7A3 | 0.68 |
| 28 | NPL-PON1 | E2RPW3 | 1 | 69 | AAL-TF | E2QSM8 | 0.79 |
| 29 | JAC-ITIH4 | ENSCAFT00000039632 | 1 | 70 | JAC-C9 | 73954295 | 0.84 |
| 30 | EPHA-ITIH2 | F1PG39 | 1 | 71 | NPL-ITIH2 | F1PG39 | 0.68 |
| 31 | HAA-C3 | 73987236 | 1 | 72 | AAL-C4A | F1PWR2 | 0.63 |
| 32 | ConA-PLG | F1Q421 | 1 | 73 | ConA-A1BG | 73947277 | 0.74 |
| 33 | JAC-CFI | E2R7A3 | 1 | 74 | HPA-C4A | F1PWR2 | 0.79 |
| 34 | LPHA-CFB | 73972310 | 1 | 75 | MAA-APOD | E2RNL8 | 0.68 |
| 35 | WGA-C4A | F1PWR2 | 1 | 76 | MAA-C3 | 73987236 | 0.63 |
| 36 | NPL-ITIH4 | ENSCAFT00000039632 | 1 | 78 | GNL-GSN | E2QZK2 | 0.79 |
| 37 | LPHA-ITIH2 | F1PG39 | 1 | 81 | UEA-TF | E2QSM8 | 0.63 |
| 38 | SNA-COL15A1 | 73972000 | 1 | 82 | AAL-ITIH1 | F1Q418 | 0.74 |
| 39 | WFA-C3 | 73987236 | 1 | 83 | HAA-HP | P19006 | 0.68 |
| 40 | WFA-HPX | F1PZR4 | 1 | 85 | WFA-CFH | E2RK21 | 0.63 |
| 41 | WGA-QSOX1 | 73960986 | 1 | 87 | HAA-APOA1 | P02648 | 0.63 |

**Supplementary Table 1C. Candidates from GlycoSelect Group Binding Difference**


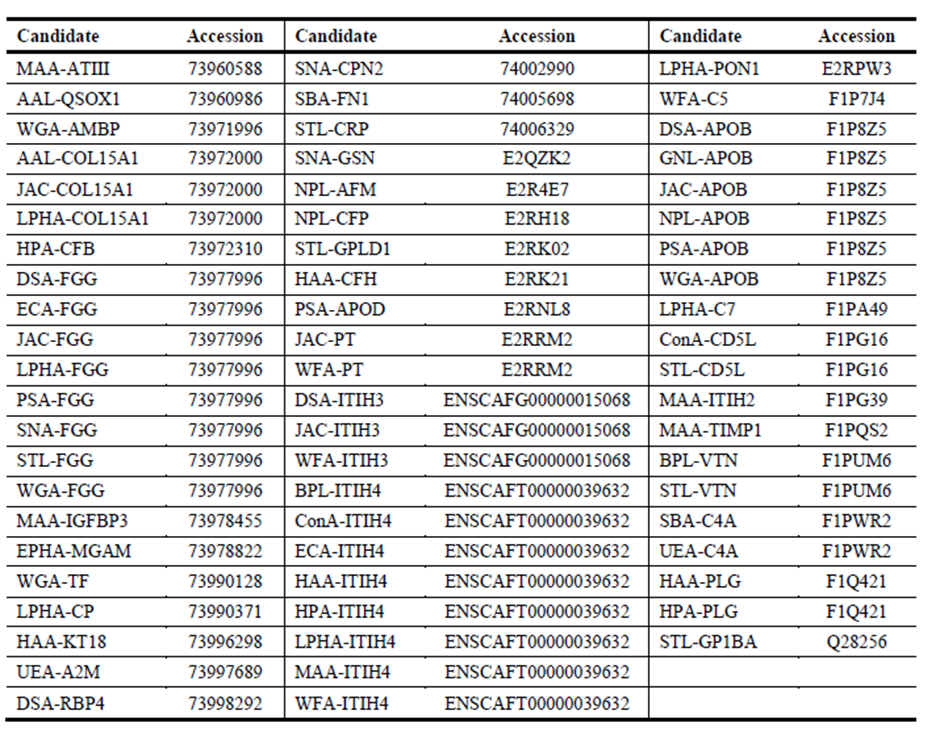


**Supplementary Table 2. (A) Case description used for biomarker validation by lectin-coupled MRM assay, and (B) proteins measured in the MRM method.**

| **A** | Sample no. | Age | Sex | Breed |
| --- | --- | --- | --- | --- |
| Normal | 1 | 14 | MN | Mixed breed |
|  | 2 | 13 | MN | Labrador Retriever cross |
|  | 3 | 8 | FS | Staffordshire Bull Terrier |
|  | 4 | 14 | FS | Bull Terrier cross |
|  | 5 | 9 | FS | Mixed breed |
|  | 6 | 10 | FS | Australian Cattle Dog cross |
|  | 7 | 6 | FS | Alaskan Malamute |
|  | 8 | 8 | FS | Labrador Retriever |
|  | 9 | 10 | MN | Golden Retriever |
|  | 10 | 13 | FI | Springer Spaniel |
|  | 11 | 11 | FS | Labrador Retriever |
|  | 12 | 12 | MN | Border Collie Cross Labrador Retriever |
|  | 13 | 10 | FS | Cocker Spaniel |
|  | 14 | 12 | FS | Australian Cattle Dog |
|  | 15 | 14 | FS | Labrador cross Kelpie |
|  | 16 | 10 | FS | Border Collie cross Labrador Retriever |
|  | 17 | 17 | MN | Border Collie cross |
|  | 18 | 11 | FS | Boxer |
|  | 19 | 9 | MN | Spaniel cross Poodle |
|  | 20 | 11 | MN | Staffordshire Bull Terrier |
|  | 21 | 9 | FS | Welsh Springer Spaniel |
|  | 22 | 10 | MN | Keeshond |
|  | 23 | 7 | FS | Rottweiler |
|  | 24 | 10 | FS | Labrador Retriever |
|  | 25 | 8 | FS | Hungarian Vizsla |
|  | 26 | 13 | FS | Australian Cattle Dog |
|  | 27 | 9 | FS | Whippet |
|  | 28 | 8 | MN | Standard Poodle |
|  | 29 | 9 | FS | German Shepherd Dog |
|  | 30 | 8 | FS | German Shepherd Dog |
| HSA-like | 1 | 11 | FS | West Highland White Terrier cross |
|  | 2 | 8 | MN | Labrador Retriever |
|  | 3 | 12 | FS | Terrier cross |
|  | 4 | 10 | FS | Labrador Retriever |
|  | 5 | 13 | FS | Beagle |
|  | 6 | 10 | FS | Staffordshire Bull Terrier |
|  | 7 | 15 | FS | Border Collie cross |
|  | 8 | 13 | MI | Australian Cattle Dog |
|  | 9 | 9 | MN | Standard Poodle |
|  | 10 | 10 | MN | American Cocker Spaniel |
|  | 11 | 12 | MN | Soft Coated Wheaten Terrier |
|  | 12 | 13 | MN | Greyhound |
|  | 13 | 10 | FS | Mixed breed |
|  | 14 | 11 | MN | Beagle |
|  | 15 | 9 | FS | Golden Retriever |
|  | 16 | 13 | MN | Airedale Terrier |
|  | 17 | 7 | FS | Mixed breed |
|  | 18 | 10 | MI | Labrador Retriever |
|  | 19 | 11 | MN | Labrador Retriever |
|  | 20 | 8 | MN | Mixed breed |
|  | 21 | 10 | FS | German Shepherd Dog |
|  | 22 | 13 | MN | Jack Russell Terrier |
|  | 23 | 12 | FS | Australian Shepherd Dog |
|  | 24 | 12 | FS | Golden Retriever |
|  | 25 | 12 | FS | Basset Hound |
|  | 26 | 9 | MN | Collie |
|  | 27 | 11 | FS | Australian Shepherd Dog |
|  | 28 | 14 | MI | Terrier cross |
|  | 29 | 9 | MN | Springer Spaniel |
|  | 30 | 8 | MI | Labrador Retriever |
|  | 31 | 13 | MN | Tibetan Spaniel |
|  | 32 | 7 | MN | Beagle |
|  | 33 | 6 | MN | Labrador Retriever |
| HSA | 1 | 11 | FS | Australian Cattle Dog cross |
|  | 2 | 15 | FS | Golden Retriever |
|  | 3 | 7 | MN | German Shepherd Dog cross |
|  | 4 | 11 | FS | Irish Setter |
|  | 5 | 10 | FS | Australian Cattle Dog |
|  | 6 | 12 | FS | Irish Setter |
|  | 7 | 7 | MN | German Shepherd Dog |
|  | 8 | 8 | FS | Whippet |
|  | 9 | 11 | MN | Cavalier King Charles Spaniel |
|  | 10 | 10 | MN | Dalmatian |
|  | 11 | 13 | MN | Rhodesian Ridgeback cross |
|  | 12 | 11 | MN | Mixed breed |
|  | 13 | 14 | FS | Stumpy Tail Australian Cattle Dog |
|  | 14 | 11 | FI | Labrador Retriever |
|  | 15 | 13 | MI | Labrador Retriever |
|  | 16 | 10 | FS | Rottweiler |
|  | 17 | 9 | MN | Labrador Retriever |
|  | 18 | 10 | FS | Rottweiler |
|  | 19 | 9 | FS | German Shepherd Dog |
|  | 20 | 9 | MN | Siberian Husky |
|  | 21 | 12 | MI | Labrador Retriever |
|  | 22 | 11 | MN | Mixed breed |
|  | 23 | 13 | MN | Mixed breed |
|  | 24 | 12 | MN | Miniature Schnauzer |
|  | 25 | 10 | FS | Mixed breed |
|  | 26 | 9 | MN | Golden Retriever |
|  | 27 | 10 | FI | Mixed breed |
|  | 28 | 9 | FS | Australian Shepherd Dog |
|  | 29 | 10 | MN | Mastiff |
|  | 30 | 4 | MN | Great Dane |
|  | 31 | 12 | MI | Golden Retriever |
|  | 32 | 12 | FS | Mixed breed |
|  | 33 | 11 | FS | Mixed breed |
|  | 34 | 13 | FI | Australian Cattle Dog cross |
|  | 35 | 10 | MN | Golden Retriever |
|  | 36 | 8 | MN | Alaskan Malamute |
|  | 37 | 10 | MN | Golden Retriever |
|  | 38 | 8 | FS | Flat-Coated Retriever |
|  | 39 | 8 | MN | Akita |
|  | 40 | 13 | MN | Miniature Schnauzer |
|  | 41 | 11 | FS | Rottweiler |
|  | 42 | 11 | MI | Golden Retriever |
|  | 43 | 12 | FS | Mixed breed |
|  | 44 | 11 | MN | Labrador Retriever |
|  | 45 | 13 | FS | Golden Retriever |
|  | 46 | 11 | FS | Mixed breed |
|  | 47 | 8 | FS | Golden Retriever |
|  | 48 | 11 | MN | Golden Retriever |
|  | 49 | 11 | FS | Shetland Sheep Dog |
|  | 50 | 12 | FS | Mixed breed |
|  | 51 | 11 | FS | Mixed breed |
|  | 52 | 15 | MN | Bichon Frisé |
|  | 53 | 14 | MI | Australian Shepherd Dog |
|  | 54 | 9 | MN | Irish Setter |
|  | 55 | 8 | MN | Scottish Terrier |

**(B) Proteins and peptides measured in the MRM method**

| Protein | Peptide sequence |
| --- | --- |
| A1AT | AVLTIDEK |
| A1AT | IAPNLADFAFSLYR |
| A1AT | LSISGTYDLK |
| A1BG | DTAPPFAGSAPSSSLELR |
| A1BG | FPLGAVTSDTR |
| A1BG | GVTYLLR |
| A2M | AVDQSVLLAKPEAELSAASVYNLLPVK |
| A2M | LLIYAILPDGEVVGDSAK |
| A2M | YSNPSNCYSGESQAVCEK |
| AFM | ADAGFLPPLPTLDPEEK |
| AFM | FLVNLVK |
| AFM | LANDVLLENICALEGLPQK |
| AHSG | AQLTPLPPSTYVEFAVAATDCVAAEVTDPAK |
| AHSG | ELDCDDPETEQAALVAVDYINNHVLR |
| AHSG | QDGQFTVLFAK |
| AHSG | VVQPDVAVAAGPVVHPCPGR |
| AMBP | EGFGHLSPTGTTEFWLGNEK |
| AMBP | IHLISTQSAIPYALR |
| AMBP | LTIGEGQQHHLGGAK |
| AMBP | TVAACSLPIVPGPCR |
| AMPEP | DLVSTLFAEWR |
| AMPEP | SEVYGPMK |
| ANGP1 | DADNDNCMCK |
| ANGP1 | ESTTDQYNTNALQR |
| ANGP1 | IENYIVENM[+147.0]K |
| ANGPT4 | DADNDNCLCK |
| ANGPT4 | VVAQGPASVR |
| APOB | GFEPTLEALFGK |
| APOB | LNGEIQALELPQK |
| APOB | LTLDIQNK |
| APOH | FTCPLTGVWPTNTVR |
| APOH | TFYDPGEQIAYTCQPGYVFR |
| APOH | VCPFAGILENGAVR |
| APOH | VTCPPPSVPK |
| ATIII | FATAFYQHVADSK |
| ATIII | TSDQVHFFFAK |
| ATIII | VAEGTQVLELPFK |
| C3 | GVFVLNK |
| C3 | VFTVDHNLLPIGR |
| C3 | VGLVAVDK |
| C4A | ALEVLQEEDLIDEDDIPVR |
| C4A | SFFPENWLWTLEK |
| C4A | STQDTVIALDALSAYWIVSHTTK |
| C4BPA | CHFGYEPASDGPTTLTCQK |
| C4BPA | HSGGDEDFYTYGSSVTYR |
| C4BPA | SSTLTCLDDTWNYSEFCTK |
| C5 | ALVEGVDQLLTDYQIK |
| C5 | DVLDQLVGEVPVTLSAHSVNVNQEVSDLESK |
| C5 | IDTQDIEASSFR |
| C5 | VFQALETSDLGCGAGGGR |
| C7 | ESSPCEDEELQHLR |
| C7 | LIDQYGTHYLQSGSLGGEYK |
| C7 | LQLDNPAGNK |
| C7 | LTPLYELVK |
| CD5L | EISLQDCPSEVWEK |
| CD5L | IWLDDVACSGNER |
| CD5L | YGEWGTICDDGWDIK |
| CFB | FIQVGVISWGVVDVCK |
| CFB | FLCTGGVNPYADPNTCK |
| CFB | HAIILLTDGK |
| CFH | LASIPLESDQEYTFGNVVR |
| CFH | SFYYHCDEDFVTPSER |
| CFH | VVYTCNEGYQLIGR |
| CFI | ECSLPHSIPACVPWSSYLFQPNDR |
| CFI | SFECLRPGAK |
| CFI | VANYFDWISHHVGR |
| CFP | HCYNIQHCDWK |
| CFP | IPVSFEK |
| CFP | MCNHPAPQHGGPPCAGDAVR |
| COL15A1 | 2VVWHGSSTHGVR |
| COL15A1 | ADFQCFQQAR |
| COL15A1 | VVWHGSSTHGVR |
| CP | AAAGIEDSACIPWAYYSTVDQVK |
| CP | DIASGLIGPLIVCR |
| CP | IYHSHIDAPK |
| CPN2 | DHLGFQAPGPGHGDPGSSWDLGVEER |
| CPN2 | ELFLDGNSISELPPGVFSR |
| CPN2 | VVFLNAPLQHLGPDAFGGLPR |
| CRP | AFVFPR |
| CRP | ALSPNVLNWR |
| CRP | SQSNEILLFK |
| CSPG4 | GPVPSCLR |
| CSPG4 | VLEQPLHGALR |
| EPHA1 | NEDYMVHVR |
| EPHA1 | WQCNQPSAR |
| EPHB3 | DDLLYNVICK |
| EPHB3 | EIDVSCVK |
| EPHB3 | ESQCRPCPPGSYK |
| EREG | IAYSLLGLK |
| EREG | NLPATDPIQR |
| EREG | VTGVVTQGASR |
| FLT1 | ILTHIGHHLNVVNLLGACTK |
| FLT1 | VTEEDEGVYHCK |
| FN1 | SSPVVIDASTAIDAPSNLR |
| FN1 | TFYSCTTEGR |
| FN1 | VPGTSASATLTGLTR |
| FN1 | VTWAPPPSIELTNFLVR |
| GP1BA | GQDLLGTVGVR |
| GP1BA | NVPVHTYQGK |
| GP1BA | SSLFLWVR |
| GPLD1 | AQYVLVSPEASSR |
| GPLD1 | NQVVAAAGR |
| GPLD1 | TLLLVGSPTWK |
| GSN | AGALNSNDAFVLK |
| GSN | EVQGFESATFLGYFK |
| GSN | HTQVSVLPEGGETPLFK |
| IGFBP3 | EVEDTLNR |
| IGFBP3 | GFCWCVDK |
| IGFBP3 | YGQPLPGFDAR |
| ITGAV | AGTQLLAGLR |
| ITGAV | ASGDFQTTK |
| ITGAV | TVEYAPCR |
| ITIH2 | FLHVPDTFDGHFDGVPVISK |
| ITIH2 | FYNQVSTPLLR |
| ITIH2 | IQPSGGTNINEALLR |
| ITIH3 | EVSFDVELPK |
| ITIH3 | LTAQALDLSLK |
| ITIH3 | VSFKPSLDQQR |
| ITIH4 | AAAQEQYSAAVAR |
| ITIH4 | LALDNGGLAR |
| ITIH4 | VTFELVYEELLK |
| LBP | EGLVALQK |
| LBP | LADGFPLPLLK |
| LBP | SFRPLVPK |
| LRG1 | ILDLSNNQLK |
| LRG1 | LAAVAAGAFR |
| LRG1 | YLFLSDNK |
| MGAM | GFPEFVK |
| MGAM | GVEDDVFIK |
| MGAM | STFPSSGR |
| MMP19 | LFTGVPDQPSAAVSWR |
| MMP19 | QSPYCSNSFDGPGR |
| MMP19 | TFQEASELPVSGQLDDATR |
| ORM1 | DLCGPVEK |
| ORM1 | ENFGILLLTK |
| ORM1 | GLSLYTR |
| OVA | DILNQITKPNDVYSFSLASR |
| OVA | GGLEPINFQTAADQAR |
| OVA | ISQAVHAAHAEINEAGR |
| OVA | YPILPEYLQCVK |
| PLG | CTTPPPPSGPTYQCLK |
| PLG | EVNLESDVQEIEVYK |
| PLG | HFCGGTLISPEWVLTAAHCLER |
| PLG | HSIFTPETNPQAGLEK |
| PON1 | EVTPVELPNCNLVK |
| PON1 | FDEYSFNPHGISTFTDEDNTVYLLVVNHPDFK |
| PON1 | IQNILTEEPK |
| PT | LAVTTHGSPCLAWASR |
| PT | SGIECQLWR |
| PT | TFGAGEADCGLRPLFEK |
| QSOX1 | DWRPALNLAALDCADETNNAVCR |
| QSOX1 | FGVTDFPSCYLLFR |
| QSOX1 | GSPVWDLDNTLR |
| QSOX1 | NDDEYLALIFEK |
| QSOX1 | VLNTEGDVVSK |
| RBP4 | GNDDHWIIDTDYDTYAVQYSCR |
| RBP4 | LLNLDGTCADSYSFVFSR |
| RBP4 | YWGVASFLQK |
| SEPP1 | DDFLIYDR |
| SEPP1 | EGFLNISYVVVNHQGLSSQLK |
| SEPP1 | QPPAWSIR |
| SERPING1 | GFTSVSQIFHSPDLPIR |
| SERPING1 | LYHAFSAVK |
| SERPING1 | NLENLLSYPEDFACVHQALK |
| SERPING1 | TSLEPFYLK |
| TF | CLQDGVGDVAFLR |
| TF | DLLFTDATDGFLR |
| TF | EDLIWELLNQAQEHFGK |
| TF | LCQLCVGEGTDK |
| TIE2 | FAYAGIDCSAEEAA |
| TIE2 | FQDVIGEGNFGQVLK |
| TIE2 | NILVGENYVAK |
| TIMP1 | EPGICTWQSLRPR |
| TIMP1 | EPGLCTWQSLR |
| TIMP1 | GFSALGNASDIR |
| VEGFC | FAAAHYNAEILK |
| VEGFC | HCEQGLIFSEEVCR |
| VEGFC | TCPTNYIWNNHLCR |
| VTN | DVWGIEGPIDAAFTR |
| VTN | FEDGVLDPDYPR |
| VTN | GIPDNVDAAFALPAR |
| VTN | GQYCYELDEK |

**Supplementary Tables 3A-C: Filtering of the LeMBA-MRM-MS dataset.**

| 3A. |  | Group |  |
| --- | --- | --- | --- |
| Site | **HSA** | **Non-HSA** | **Normal** |
| Missing | 0 | 1 | 0 |
| BVSC | 1 | 3 | 0 |
| CSU | 40 | 18 | 0 |
| EHS | 0 | 1 | 0 |
| QVS | 7 | 4 | 0 |
| UQ | 2 | 8 | 30 |
| Total | **50** | **35** | **30** |

**3B.**

| Number of Proteins |  | After filtering |  | Before filtering |  |
| --- | --- | --- | --- | --- | --- |
| Peptide per protein | **3** | **2** | **1** | **Number of proteins** | **Number of peptides** |
| AAL | 3 | 4 | 19 | 58 | 176 |
| DSA | 8 | 6 | 23 | 58 | 179 |
| LPHA | 1 | 10 | 15 | 59 | 182 |
| NPL | 7 | 14 | 20 | 59 | 182 |
| PSA | 7 | 11 | 19 | 58 | 180 |
| SNA | 2 | 7 | 18 | 58 | 181 |
| WDA | 9 | 8 | 20 | 58 | 181 |

**3C.**

| SITE |  | AAL | DSA | LPHA | NPL | PSA | SNA | WGA |
| --- | --- | --- | --- | --- | --- | --- | --- | --- |
| BVSC | **Remaining** | 3 | 4 | 3 | 2 | 4 | 4 | 2 |
|  | **Removed** | 1 | 0 | 1 | 2 | 0 | 0 | 2 |
| CSU | **Remaining** | 47 | 47 | 47 | 41 | 48 | 49 | 48 |
|  | **Removed** | 11 | 11 | 11 | 17 | 10 | 9 | 10 |
| EHS | **Remaining** | 1 | 1 | 1 | 1 | 1 | 1 | 1 |
|  | **Removed** | 0 | 0 | 0 | 0 | 0 | 0 | 0 |
| MISSING | **Remaining** | 1 | 1 | 1 | 1 | 1 | 1 | 1 |
|  | **Removed** | 0 | 0 | 0 | 0 | 0 | 0 | 0 |
| QVS | **Remaining** | 9 | 8 | 6 | 6 | 10 | 8 | 6 |
|  | **Removed** | 2 | 3 | 5 | 5 | 1 | 3 | 5 |
| UQ | **Remaining** | 29 | 29 | 26 | 33 | 34 | 24 | 37 |
|  | **Removed** | 11 | 11 | 14 | 7 | 6 | 16 | 3 |

**Supplementary Table 4. Patients profile summary for samples used in lectin/immunohistochemistry.**

| **Diagnosis** | **Breed** | **Sex** | **Age** | **Organ** | **Growth**  **Pattern** | **Grade** |
| --- | --- | --- | --- | --- | --- | --- |
| HSA | Australian Cattle Dog | NA* | NA | spleen | solid | 3 |
| HSA | Beagle | MS | 7 | spleen | solid | 1 |
| HSA | Border Collie | MS | 9 | sternal mass | solid | 3 |
| HSA | Bull Mastiff | F | NA | spleen | solid | 2 |
| HSA | Bull terrier | NA | 14 | spleen | mixed | 3 |
| HSA | Crossbred | M | 6 | spleen | typical | 3 |
| HSA | Crossbred | M | 12 | spleen | typical | 3 |
| HSA | Crossbred | MS | 11 | spleen | mixed | 3 |
| HSA | Crossbred | NA | NA | spleen | mixed | 2 |
| HSA | Crossbred | NA | NA | spleen | mixed | 3 |
| HSA | Crossbred | F | 13 | spleen | typical | 2 |
| HSA | Curly Retriever | M | 12 | heart | typical | 2 |
| HSA | Curly Retriever | NA | 12 | spleen | mixed | 1 |
| HSA | Dachshund | FS | 6 | spleen | mixed | 2 |
| HSA | Dalmatian | MS | 9 | spleen | mixed | 3 |
| HSA | German Shepherd | MS | 7 | spleen | typical | 2 |
| HSA | German Shepherd | M | 11 | peritoneal  mass | cavernous | 1 |
| HSA | German Shepherd | MS | 13 | spleen | mixed | 3 |
| HSA | Golden Retriever | NA | 7 | spleen | mixed | 2 |
| HSA | Golden Retriever | FS | 14 | spleen | solid | 1 |
| HSA | Golden Retriever | M | 14 | spleen | mixed | 1 |
| HSA | Irish setters | F | 11 | spleen | typical | 2 |
| HSA | Irish setters | F | 12 | liver | typical | 2 |
| HSA | Maltese | FS | 11 | spleen | solid | 2 |
| HSA | NA | NA | 9 | spleen | mixed | 2 |
| HSA | NA | NA | NA | spleen | solid | 3 |
| HSA | NA | NA | NA | liver | typical | 1 |
| HSA | Rottweiler | NA | NA | spleen | mixed | 3 |
| HSA | Springer spaniel | MS | 9 | spleen | typical | 2 |
| HSA | Staffordshire bull terrier | FS | 9 | spleen | cavernous | 1 |
| HSA | Stumpy tail cattle dog | FS | 14 | spleen | typical | 2 |
| HSA | Terrier | F | 12 | spleen | mixed | 2 |
| Adenocarcinoma | German Shepherd | F | 10 | spleen | NA | NA |
| Apocrine gland tumor | German Shepherd | MS | 13 | spleen | NA | NA |
| Fibrohistiocytic nodule | Dachshund | M | 8 | spleen | NA | NA |
| Haemorrhage | Maltese | M | 6 | spleen | NA | NA |
| Haemorrhage | NA | NA | NA | spleen | NA | NA |
| Hematoma | Australian Cattle Dog | F | 11 | spleen | NA | NA |
| Hematoma | Beagle | FS | 12 | spleen | NA | NA |
| Hematoma | NA | FS | NA | spleen | NA | NA |
| Hematoma | Staffordshire bull terrier | FS | 10 | spleen | NA | NA |
| Hematoma | NA | NA | 16 | spleen | NA | NA |
| HCA** | Crossbred | NA | 13 | spleen | NA | NA |
| HCA | Jack Russell terrier | MS | NA | liver | NA | NA |
| HCA | NA | NA | NA | liver | NA | NA |
| HCA | NA | NA | NA | liver | NA | NA |
| HCA | West HighlanAd terrier | FS | 10 | liver | NA | NA |
| Lymphoid hyperplasia | Golden Retriever | FS | 14 | spleen | NA | NA |
| Lymphoma | Labrador Retriever | MS | NA | spleen | NA | NA |
| Lymphoma | Rhodesian ridgeback | M | 12 | spleen | NA | NA |
| Lymphoma | Staffordshire bull terrier | FS | 11 | spleen | NA | NA |
| Nodular hyperplasia | Maltese | FS | 14 | spleen | NA | NA |
| Normal spleen | Crossbred | M | 13 | spleen | NA | NA |
| Normal spleen | French bulldog | NA | NA | spleen | NA | NA |
| Normal spleen | Terrier | M | NA | spleen | NA | NA |
| Ambiguous¶ | Labrador Retriever | MS | 9 | spleen | NA | NA |
| Ambiguous | NA | NA | NA | spleen | NA | NA |
| Splenitis | Crossbred | M | 8 | spleen | NA | NA |

*NA = not applicable/not available; **HCA = hepatocellular carcinoma; ¶ neoplasia samples for which pathologist could not arrive at a consensus diagnosis.

**Supplementary Table 5.** Lectins and antibodies used in the study and their antigenic retrieval (AR) methods and dilutions.

| **Lectins and Antibodies** | **Class** | **Host** | **No AR** | **Citrate Buffer**  pH 6 HIER  20 min. 90^o^ C | **Tris EDTA**  pH 9 HIER  20 min. 90^o^ C | **Proteinase K**    15 min.  Room Temp |
| --- | --- | --- | --- | --- | --- | --- |
| **DSA**  (GlcNAc)_2-4_ | NA | NA | 1:200 | - | - | - |
| **WGA**  GlcNAc | NA | NA | 1:500 | - | - | - |
| **SNA**  Neu5Acα6Gal/GalNAc | NA | NA | × | (1:50)* | 1:50 | × |
| **PSA^#^**  αMan/ αGlc | NA | NA | × | × | × | × |
| **MGAM^#^**  (Clone: HPA002270) | Polyclonal | Rabbit | × | × | × | × |
| **C7**  (Clone: ab192346) | Polyclonal | Rabbit | - | 1:75 | - | - |
| **Vitronectin**  (Clone: PA5-27909) | Polyclonal | Rabbit | × | 1:100 | (1:100)* | × |
| **CD31**  (Clone: JC70A) | Monoclonal | Mouse | × | × | (1:20)* | 1:10 |
| **MAC387**  (Clone: MAC 387) | Monoclonal | Mouse | - | - | - | 1:300 |

× = no lectin/immunohistochemical reaction signal detected. PSA and MGAM subsequently not pursued. - = not performed. * = The dilution shown within the parenthesis was performed for optimization, but not on the samples.

**Supplementary Table 6**. Average H-scores for lectin/immunohistochemical signal intensities of each component of the control sample. The control slides were included in every batch of the experiments. The Coefficient of Variations (CV, %) were calculated to assess the variations among the repetitive runs.

| **Antibodies/Lectins** | **CD31** | **DSA** | **WGA** | **SNA** | **VTN** | **C7** |
| --- | --- | --- | --- | --- | --- | --- |
| Repeats | 5 | 9 | 11 | 9 | 8 | 8 |
| **HSA**  Mean H-score  CV (%) | NA* | 188.3  (28.1) | 219.1  (22.7) | 115.5  (81.3) | 40.3  (105.6) | 253  (9.7) |
| Positive HSA cells | NA* | 83.3 % | 74.6 % | 59.4 % | 34 % | 86.9 % |
| **Arterial endothelium**  Mean H-score  CV (%) | 280.8  (8.4) | 4.4  (95.4) | 245  (11.2) | 81.1  (49.9) | 21.4  (135.2) | 92.5  (53.8) |
| Positive endothelial cells | 93.6 % | 4.4 % | 86.1 % | 54.4 % | 21.4 % | 63.8 % |
| **Venous endothelium**  Mean H-score  CV (%) | 275.4  (4.4) | 247.2  (19.9) | 29.4  (138.6) | 87.2  (77.2) | 39.5  (130.7) | 154.4  (48.5) |
| Positive endothelial cells | 91.8 % | 83.9 % | 18.3 % | 45 % | 20.8 % | 66.9 % |

NA = not applicable. For CD31 antibody, baboon tissue (courtesy of Dr. Penny Farrell, Univ. of Sydney, with approval from the SSWAHS Animal Welfare Committee) was used for positive control to optimize immunohistochemistry protocol on dog tissues.

**Supplementary Table 7**. Results of lectin-histochemistry and immunohistochemical labeling of canine spleens with HSA.

| **Tissue components** | **Antibodies and Lectins** | | |
| --- | --- | --- | --- |
| HSA samples (n = 32) | **CD31** | **DSA** | **WGA** |
| **HSA** |  |  |  |
| Positive samples | 25/29 | 32/32 | 29/31 |
| Positive cells (%) | 43.7 | 78.7 | 69 |
| H-score (Mean ± SD) | 123.7 ± 109.8 | 171.3 ± 76.7 | 157.9 ± 109.2 |
| H-score (Median ± MAD) | 180 ± 133.4 | 180 ± 89 | 150 ± 148.3 |
| Inter Quartile Range | 215 | 135 | 197.5 |
| **Arterial endothelium** |  |  |  |
| Positive samples | 27/28 | 24/27 | 28/28 |
| Positive cells (%) | 52.68 | 30 | 73.6 |
| H-score (Mean ± SD) | 134.8 ± 87.5 | 46.9 ± 58.9 | 194.6 ± 78.5 |
| H-score (Median ± MAD) | 140 ± 126 | 26 ± 29.7 | 215 ± 70.4 |
| Inter Quartile Range | 167.5 | 60 | 110 |
| **Venous endothelium** |  |  |  |
| Positive samples | 19/26 | 26/27 | 24/28 |
| Positive cells (%) | 13.7 | 65.7 | 15.1 |
| H-score (Mean ± SD) | 37.1 ± 33.4 | 172.9 ± 59.3 | 29.4 ± 13.3 |
| H-score (Median ± MAD) | 22.5 ± 41.3 | 190 ± 81.3 | 10 ± 60.4 |
| Inter Quartile Range | 52.5 | 100 | 19 |
| **Tissue components** | **Antibodies and Lectins** | | |
| HSA samples (n = 32) | **SNA** | **VTN** | **C7** |
| **HSA** |  |  |  |
| Positive samples | 24/30 | 25/30 | 26/28 |
| Positive cells (%) | 41 | 45.5 | 68.3 |
| H-score (Mean ± SD) | 78.8 ± 91.2 | 57.4 ± 60.4 | 175.8 ± 102.6 |
| H-score (Median ± MAD) | 20 ± 29.7 | 45.1 ± 62.5 | 220 ± 103.8 |
| Inter Quartile Range | 125.75 | 82.5 | 192.5 |
| **Arterial endothelium** |  |  |  |
| Positive samples | 26/26 | 18/25 | 26/26 |
| Positive cells (%) | 53.3 | 38.7 | 65.9 |
| H-score (Mean ± SD) | 88.7 ± 68.9 | 38.7 ± 35.3 | 83.9 ± 45.6 |
| H-score (Median ± MAD) | 65 ± 66.7 | 30 ± 44.5 | 77.5 ± 24.1 |
| Inter Quartile Range | 106.25 | 75 | 26.9 |
| **Venous endothelium** |  |  |  |
| Positive samples | 16/25 | 19/28 | 26/28 |
| Positive cells (%) | 26 | 37.2 | 62.7 |
| H-score (Mean ± SD) | 52 ± 14.8 | 65.7 ± 51.5 | 139 ± 118.6 |
| H-score (Median ± MAD) | 10 ± 60.4 | 75 ± 76.2 | 25 ± 90.5 |
| Inter Quartile Range | 60 | 60 | 150 |

**Supplementary Table 8**. Results of lectin-histochemistry and immunohistochemical labelling of canine non-HSA samples.

| **Tissue components** | **Antibodies and Lectins** | | |
| --- | --- | --- | --- |
| non-HSA samples (n = 26) | **CD31** | **DSA** | **WGA** |
| **Arterial endothelium** |  |  |  |
| Positive samples | 12/12 | 18/19 | 22/23 |
| Positive cells (%) | 63.8 | 33 | 72.4 |
| H-score (Mean ± SD) | 191.3 ± 52.01 | 38 ± 30.5 | 175.4 ± 75 |
| H-score (Median ± MAD) | 195 ± 44.5 | 25 ± 37.1 | 180 ± 66.7 |
| Inter Quartile Range | 52.5 | 52.5 | 95 |
| **Venous endothelium** |  |  |  |
| Positive samples | 7/11 | 19/19 | 18/24 |
| Positive cells (%) | 7.4 | 67.6 | 21.3 |
| H-score (Mean ± SD) | 18.3 ± 35 | 187.1 ± 87.1 | 40 ± 66.5 |
| H-score (Median ± MAD) | 10 ± 14.8 | 230 ± 44.5 | 5 ± 7.4 |
| Inter Quartile Range | 15 | 85 | 36.8 |
| **Tissue components** | **Antibodies and Lectins** | | |
| non-HSA samples (n = 26) | **SNA** | **VTN** | **C7** |
| **Arterial endothelium** |  |  |  |
| Positive samples | 21/21 | 17/22 | 23/24 |
| Positive cells (%) | 58.3 | 38.4 | 50 |
| H-score (Mean ± SD) | 79.5 ± 43.2 | 43.9 ± 41.7 | 71.3 ± 58.0 |
| H-score (Median ± MAD) | 75 ± 22.2 | 35 ± 52 | 50 ± 44.5 |
| Inter Quartile Range | 20 | 52.5 | 52.5 |
| **Venous endothelium** |  |  |  |
| Positive samples | 17/22 | 14/22 | 20/22 |
| Positive cells (%) | 23.9 | 38 | 50.2 |
| H-score (Mean ± SD) | 33 ± 39.2 | 82.5 ± 97.8 | 116.8 ± 98.6 |
| H-score (Median ± MAD) | 25 ±36.3 | 35 ± 52 | 85 ± 96.4 |
| Inter Quartile Range | 45.5 | 147.5 | 182.5 |

**Supplementary Table 9.** Overall H-scores assessed from lectin/immunohistochemistry with DSA-*Datura stramonium,* WGA-Wheat Germ Agglutinin and SNA*-Sambucus nigra* lectins and anti-human CD31, anti-human VTN and anti-human complement C7 antibodies in three histopathological grades.

| **Antibodies/Lectins** | **CD31** | **DSA** | **WGA** |
| --- | --- | --- | --- |
| ***Grade 1*** |  |  |  |
| Mean ± SD | 63 ± 115.95 | 132 ± 50.57 | 215 ± 115.57 |
| Median ± MAD | 15 ± 7.41 | 100 ± 14.83 | 270 ± 192.74 |
| IQR | 10 | 55 | 187.5 |
| ***Grade 2*** |  |  |  |
| Mean ± SD | 166.2 ± 97.83 | 175.4 ± 83.65 | 160.4 ± 114.13 |
| Median ± MAD | 210 ± 44.48 | 177.5 ± 85.25 | 170 ± 151.97 |
| IQR | 85 | 97.5 | 220 |
| ***Grade 3*** |  |  |  |
| Mean ± SD | 101 ± 110.15 | 200.6 ± 67.6 | 148.60 ± 108.89 |
| Median ± MAD | 10 ± 14.83 | 180 ± 59.30 | 120 ± 151.97 |
| IQR | 192.5 | 90 | 116.25 |
|  |  |  |  |
| **Antibodies/Lectins** | **SNA** | **VTN** | **C7** |
| ***Grade 1*** |  |  |  |
| Mean ± SD | 152 ± 126.72 | 87.2 ± 91.91 | 93.75 ± 116.08 |
| Median ± MAD | 90 ± 107.49 | 70 ± 44.48 | 45 ±103.78 |
| IQR | 190 | 64.5 | 130 |
| ***Geade 2*** |  |  |  |
| Mean ± SD | 86.54 ± 89.2 | 67.94 ±54.15 | 200.7 ± 100.25 |
| Median ± MAD | 40 ± 59.30 | 75 ± 66.53 | 245.0 ± 37.07 |
| IQR | 150 | 76.25 | 92.5 |
| ***Grade 3*** |  |  |  |
| Mean ± SD | 43 ± 60.07 | 38.18 ± 40.64 | 180.9 ± 93.22 |
| Median ± MAD | 20 ±29.7 | 30 ± 44.48 | 150 ± 133.43 |
| IQR | 70 | 82.5 | 125 |

**Supplementary Figure 1.** Microscopic images demonstrating the visualization of the glycoprotein expression in the tissues. The H-scores were calculated from the combination of lectin/immunohistochemical reaction signal intensity levels and the proportion of the glycoprotein expressed cells.

| **H-score** (0-300) = [1*(% cells 1+)] + [2*(% cells 2+)] + [3*(% cells 3+)] | | |
| --- | --- | --- |
| **H-score** | **Overall intensity classification** | **Microscopic image** |
| 0 | 0 (no signal detected) | 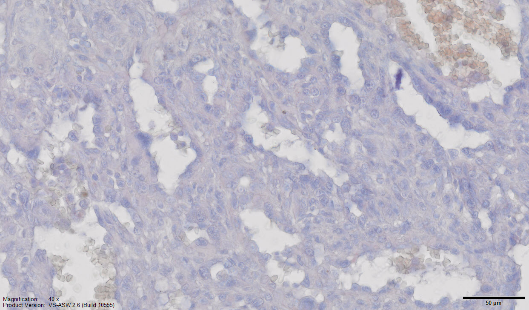 |
| 1-100 | 1 (mild) | 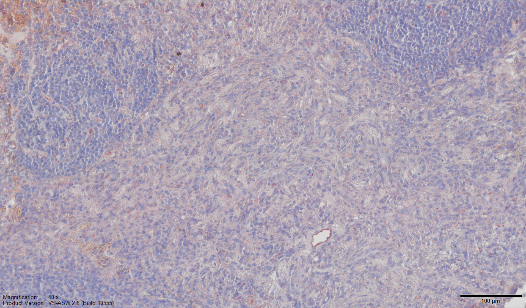 |
| 101-200 | 2 (moderate) | 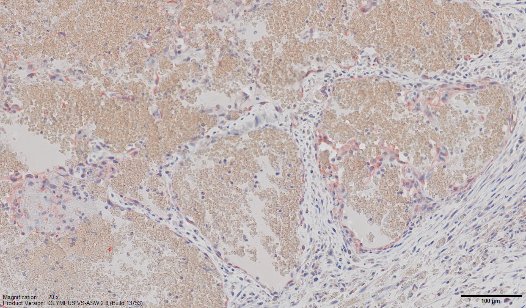 |
| 201-300 | 3 (high) | 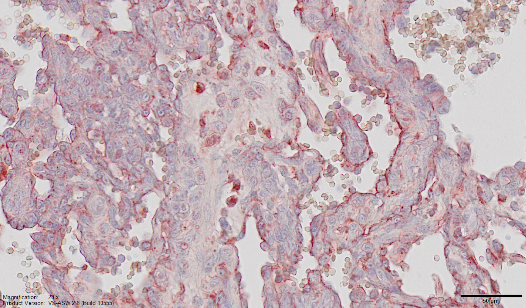 |

**Supplementary Figure 2**. Comparison of labelling patterns for lectins (DSA, WGA, SNA) and anti-CD31, anti-C7 and anti-VTN antibodies on HSA tumor cells, arterial and venous endothelial cells. Binding was visualized using AEC substrate (red) following either incubation with secondary antibody (Envision Kit) or streptavidin-horse radish peroxidase conjugate. Microphotos were generated using Aperio-scans. Original magnification 20x.

|  | Cancer | Arterial endothelium | Venous endothelium |
| --- | --- | --- | --- |
| CD31 | 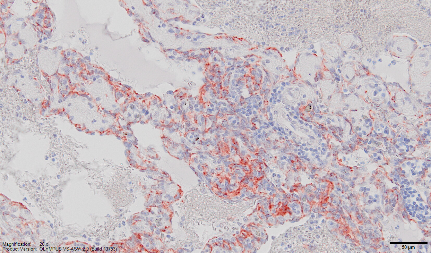 | 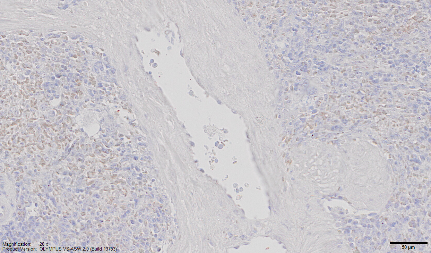 | 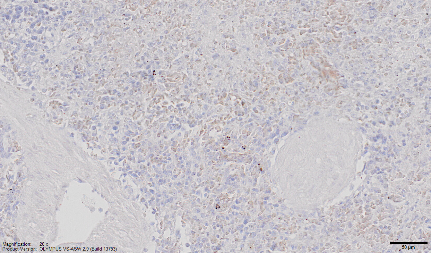 |
| DSA | 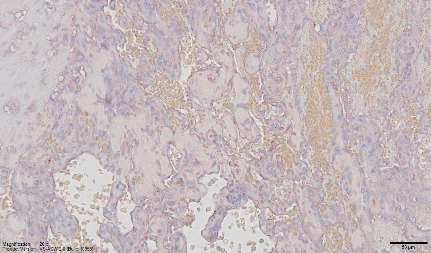 | 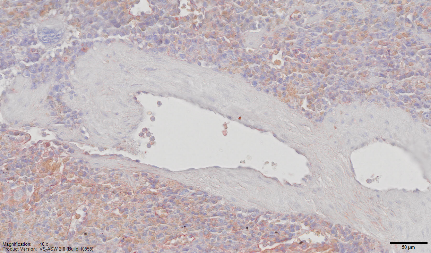 | 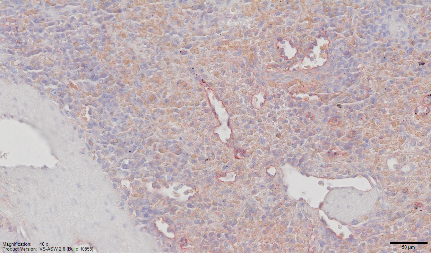 |
| WGA | 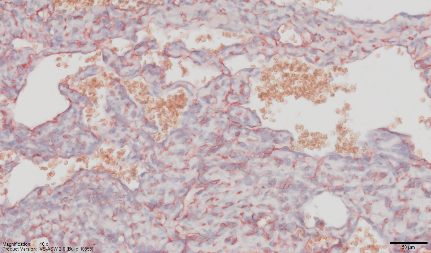 | 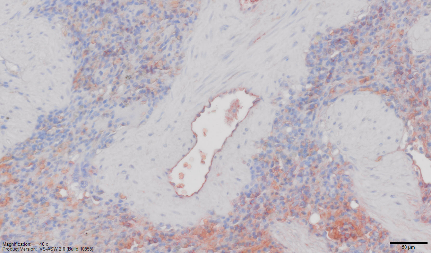 | 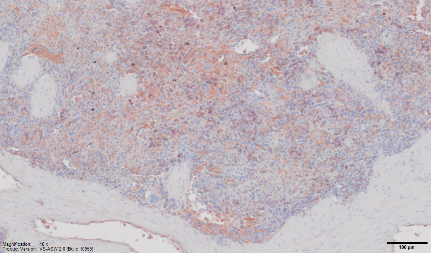 |
| SNA | 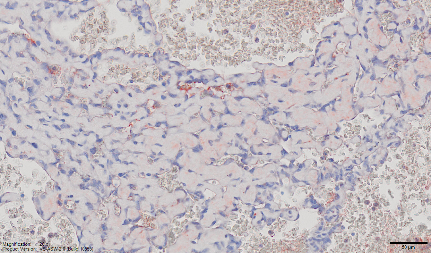 | 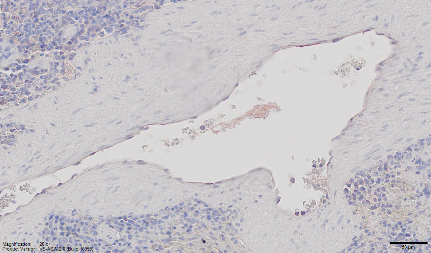 | 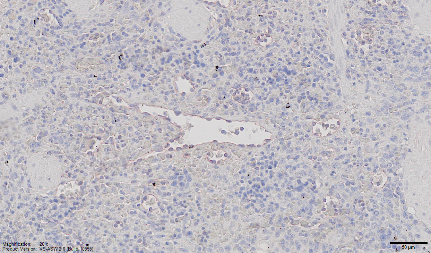 |
| VTN | 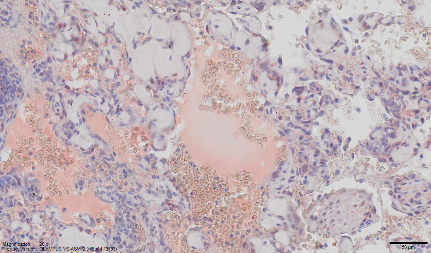 | 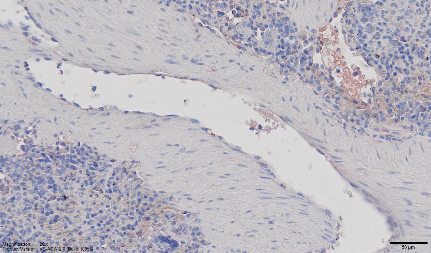 | 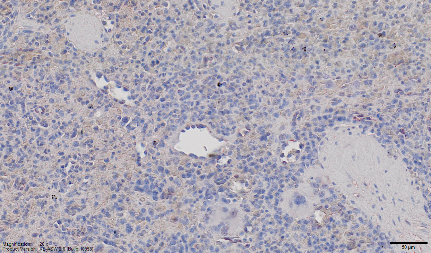 |
| C7 | 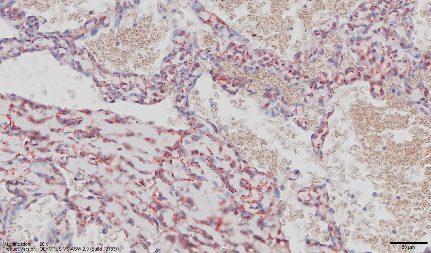 | 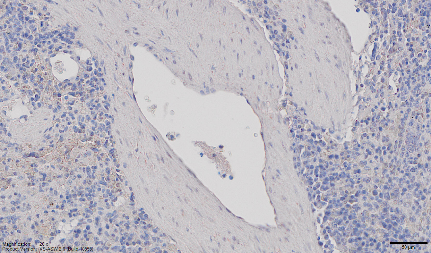 | 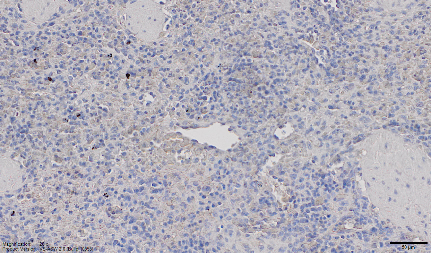 |

**Supplementary Figure 3.** Examples of immunohistochemical and lectin-histochemistry labelling of canine splenic HSA with different growth patterns. FFPE sections were reacted with anti-human CD31 antibody, anti-human VTN antibody, anti-human complement C7 antibody and biotinylated lectins DSA *(Datura stramonium),* WGA *(Wheat Germ Agglutinin)* and SNA *(Sambucus nigra)* lectins) on consecutive splenic HSA sections (cavernous, sample 18/015280E; solid, 11/065749G; typical slit-like, 12/025492A). Binding was visualized using AEC substrate (red) following either incubation with secondary antibody (Envision Kit) or streptavidin-horse radish peroxidase conjugate. Microphotos were generated using Aperio-scans. Original magnification 20x.

|  | Typical slit-like | Solid | Cavernous |
| --- | --- | --- | --- |
| CD31 | 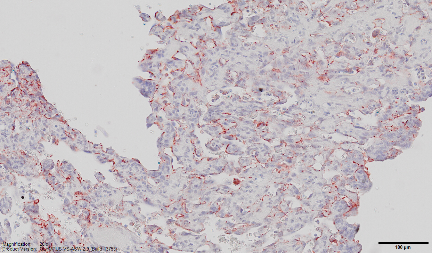 | 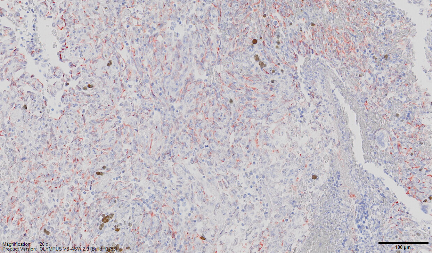 | 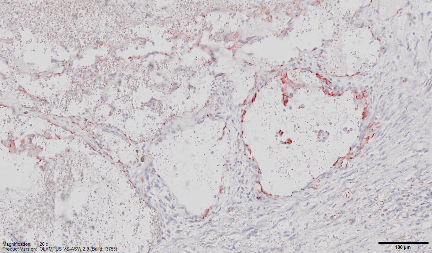 |
| DSA | 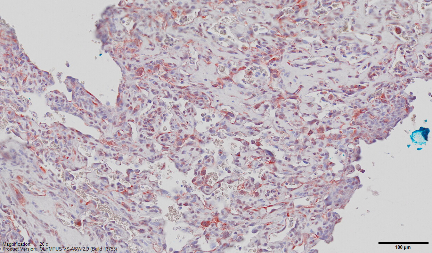 | 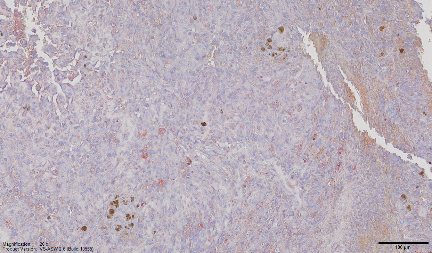 | 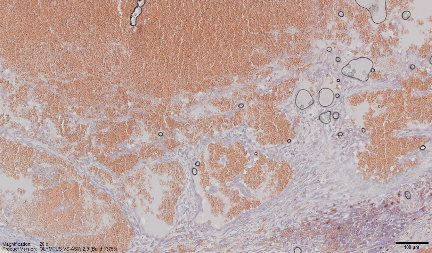 |
| WGA | 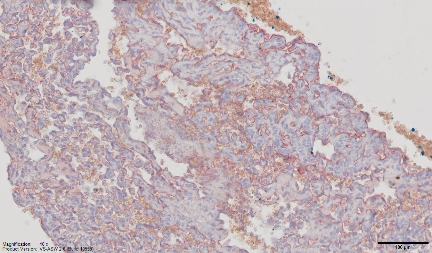 | 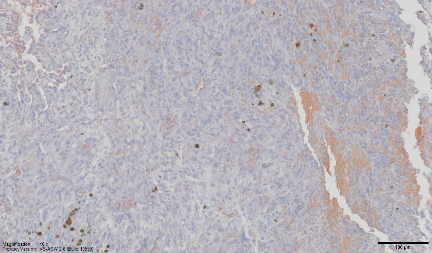 | 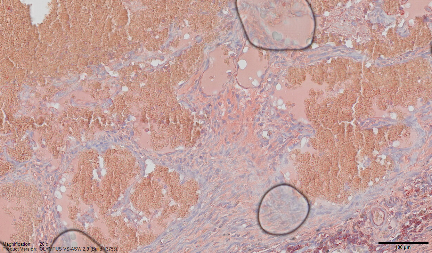 |
| SNA | 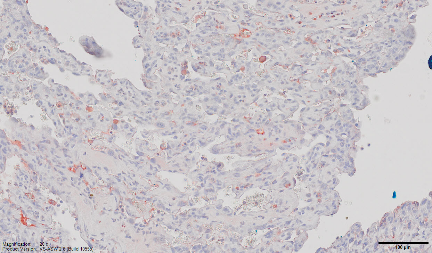 | 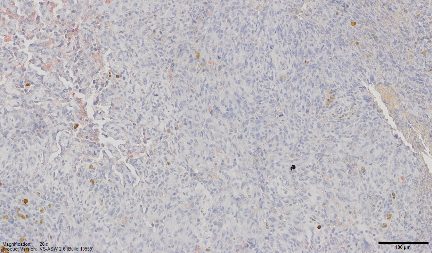 | 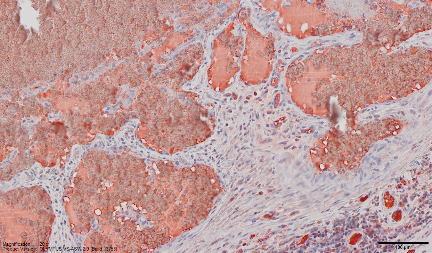 |
| VTN | 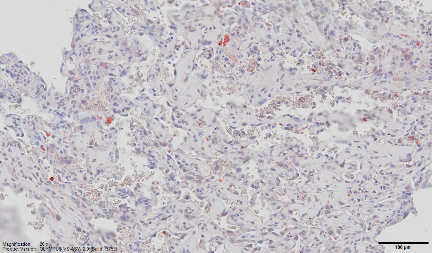 | 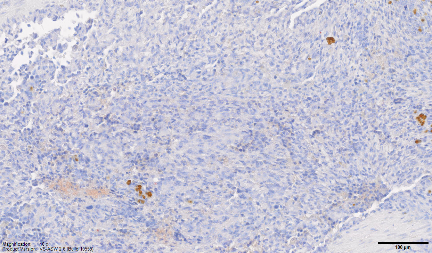 | 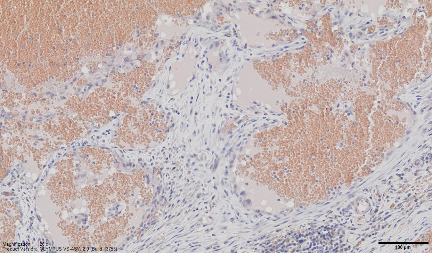 |
| C7 | 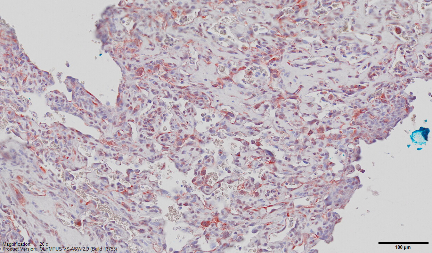 | 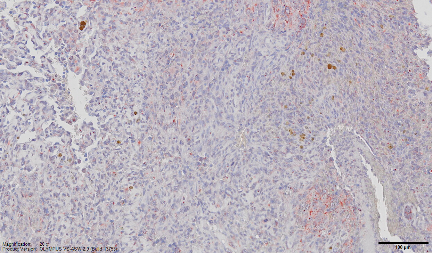 | 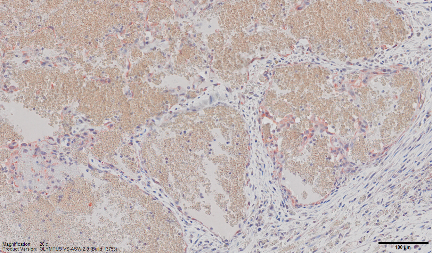 |


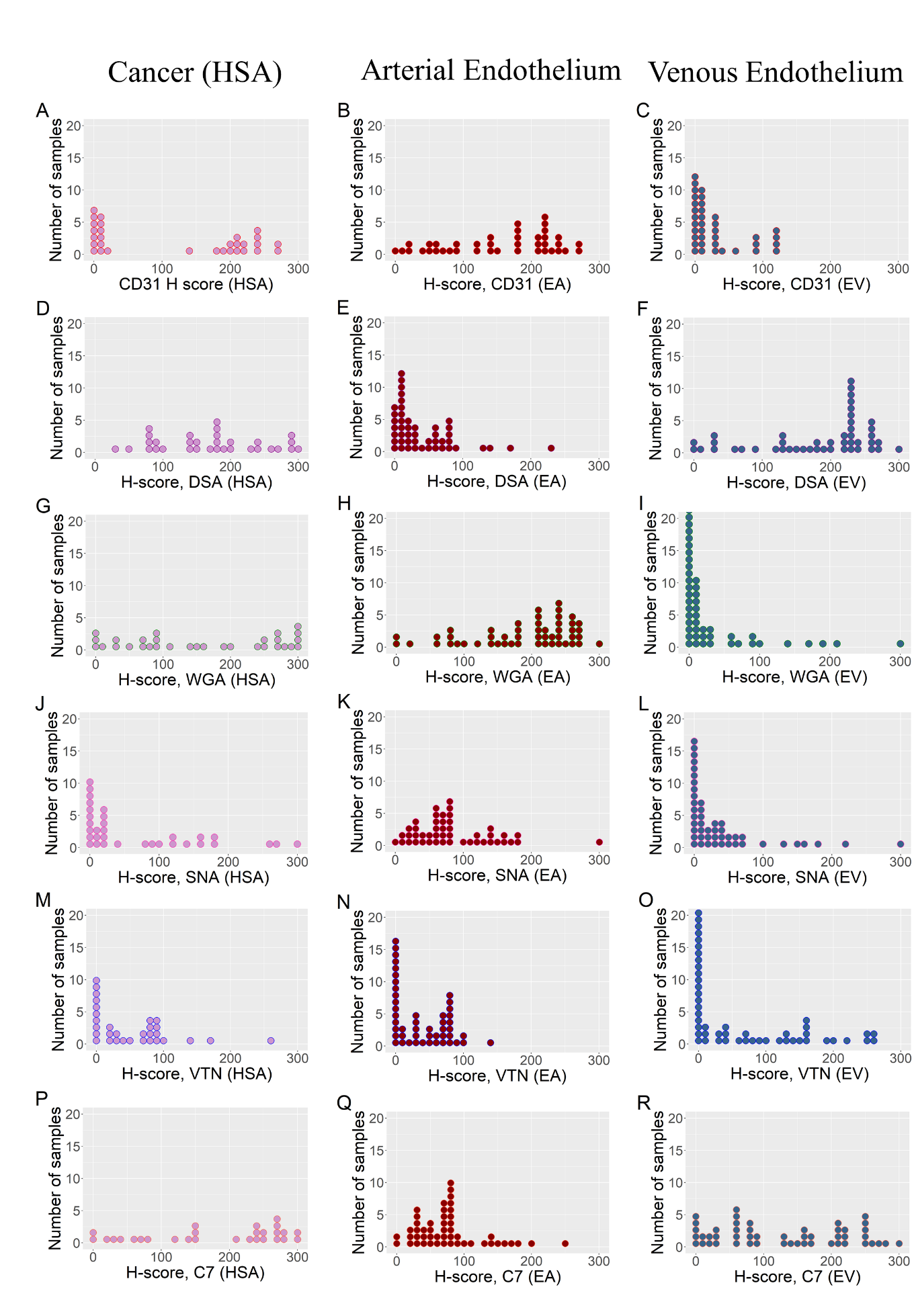
**Supplementary Figure 4.** H-score distribution of lectin- and immunohistochemical labelling of HSA cancer cells, arterial and venous endothelial cells. FFPE sections of canine splenic HSA were immunolabeled for CD31, complement C7 and VTN or reacted with the lectins DSA, WGA and or SNA. The score assessment was performed on three tissue components; HSA cancer cells, arterial endothelium and venous endothelium separately.

**Supplementary Figure 5.** Boxplots of H-score distribution, assessed by lectin/immunohistochemistry for DSA-*Datura stramonium,* WGA-Wheat Germ Agglutinin and SNA*-Sambucus nigra* lectins and anti-human CD31, anti-human VTN and anti-human complement C7 antibodies, on thirty-two HSA FFPE tissue sections. The samples were divided into three groups by histopathological grades, three tissue components were assessed separately. Trends of correlation between level of glycoprotein expression and histopathological grade were observed in DSA and SNA histochemistry.


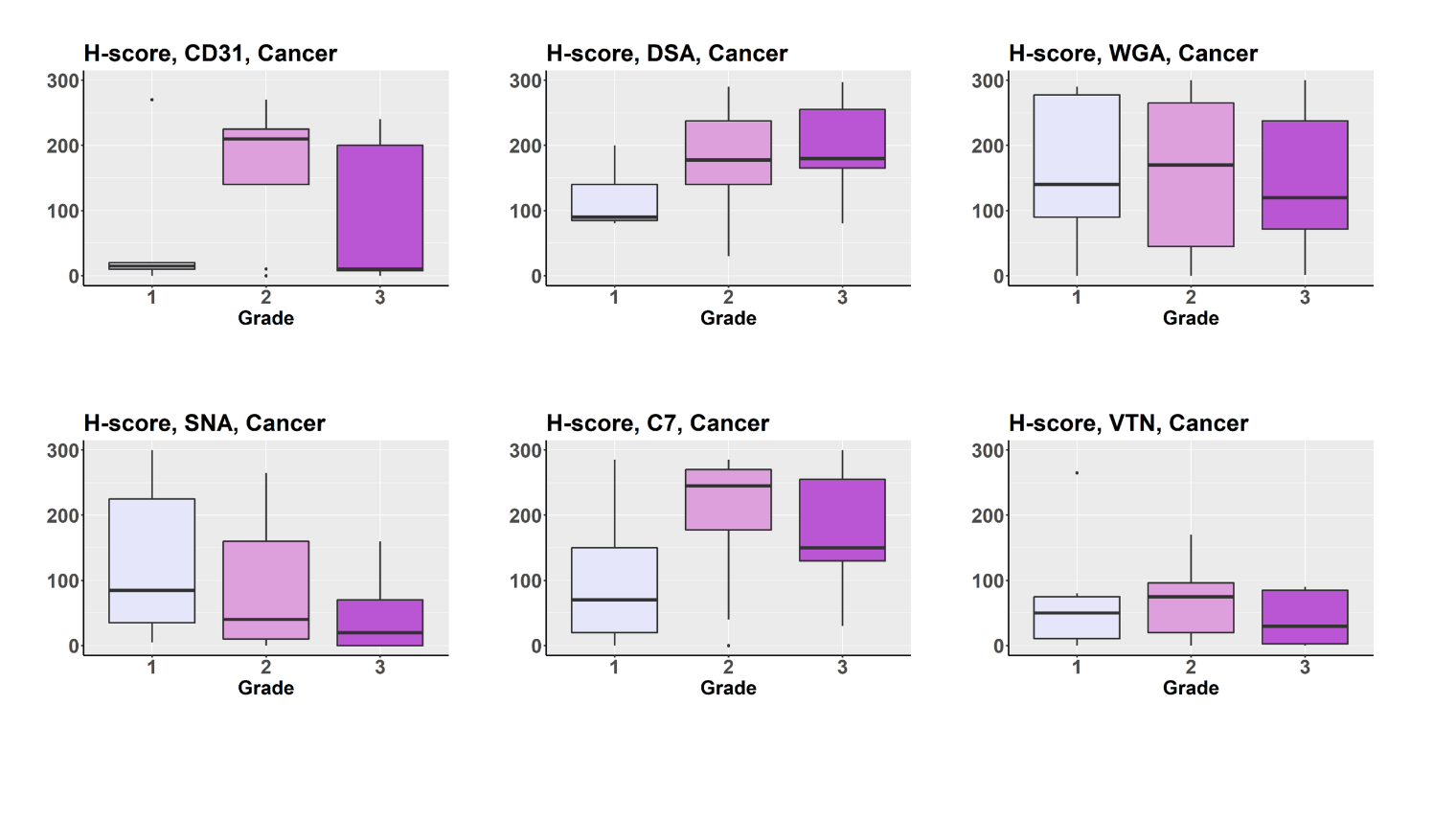


**Supplementary Figure 6.** Boxplots of H-score distribution, assessed for CD31, DSA, WGA, SNA, VTN and C7 lectin/immunohistochemical labelling signal intensity on thirty-two HSA FFPE tissue sections. No statistically difference in the signal intensity between solid or typical HSA growth pattern was found.


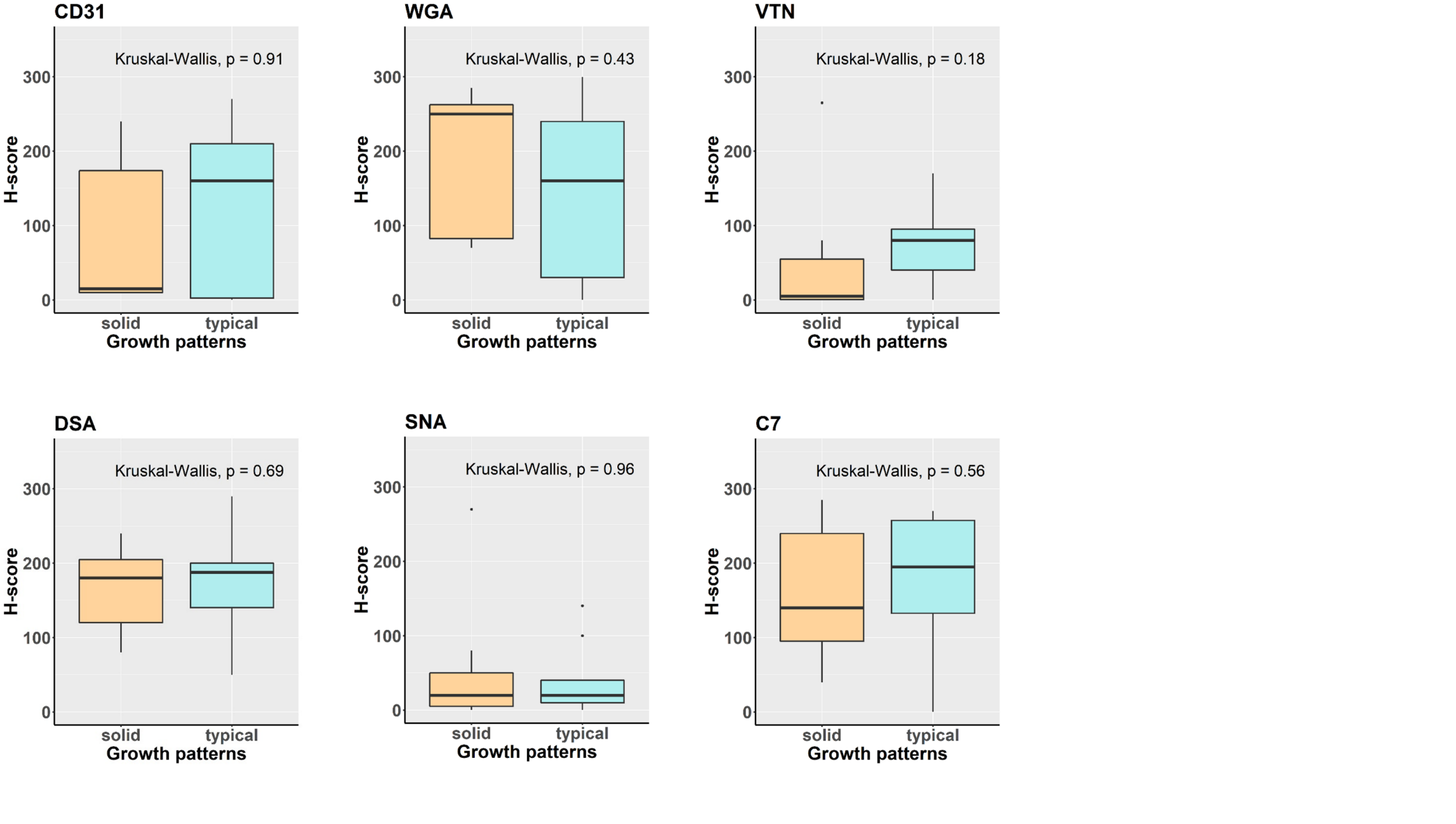

Supplement: Supplementary file 1 [file vetsci-08-00038-s001.zip › Supplementary Files_lectin validation manuscript - hbo 21022021.docx]
